# A secretomic view of woody and nonwoody lignocellulose degradation by *Pleurotus ostreatus*

**DOI:** 10.1186/s13068-016-0462-9

**Published:** 2016-02-29

**Authors:** Elena Fernández-Fueyo, Francisco J. Ruiz-Dueñas, María F. López-Lucendo, Marta Pérez-Boada, Jorge Rencoret, Ana Gutiérrez, Antonio G. Pisabarro, Lucía Ramírez, Angel T. Martínez

**Affiliations:** Department of Biotechnology, Delft University of Technology, Julianalaan 136, 2628 BL Delft, The Netherlands; Centro de Investigaciones Biológicas, CSIC, Ramiro de Maeztu 9, 28040 Madrid, Spain; Instituto de Recursos Naturales y Agrobiología de Sevilla, CSIC, PO Box 1052, 41080 Seville, Spain; Department of Agrarian Production, Universidad Pública de Navarra, 31006, Pamplona, Spain

**Keywords:** *Pleurotus ostreatus*, Secreted proteins, Poplar wood, Wheat straw, LC–MS/MS, Lignin-modifying enzymes, Laccases, Carbohydrate-active enzymes, 2D NMR

## Abstract

**Background:**

*Pleurotus ostreatus* is the second edible mushroom worldwide, and a model fungus for delignification applications, with the advantage of growing on woody and nonwoody feedstocks. Its sequenced genome is available, and this gave us the opportunity to perform proteomic studies to identify the enzymes overproduced in lignocellulose cultures.

**Results:**

Monokaryotic *P. ostreatus* (PC9) was grown with poplar wood or wheat straw as the sole C/N source and the extracellular proteins were analyzed, together with those from glucose medium. Using nano-liquid chromatography coupled to tandem mass spectrometry of whole-protein hydrolyzate, over five-hundred proteins were identified. Thirty-four percent were unique of the straw cultures, while only 15 and 6 % were unique of the glucose and poplar cultures, respectively (20 % were produced under the three conditions, and additional 19 % were shared by the two lignocellulose cultures). Semi-quantitative analysis showed oxidoreductases as the main protein type both in the poplar (39 % total abundance) and straw (31 %) secretomes, while carbohydrate-active enzymes (CAZys) were only slightly overproduced (14–16 %). Laccase 10 (LACC10) was the main protein in the two lignocellulose secretomes (10–14 %) and, together with LACC2, LACC9, LACC6, versatile peroxidase 1 (VP1), and manganese peroxidase 3 (MnP3), were strongly overproduced in the lignocellulose cultures. Seven CAZys were also among the top-50 proteins, but only CE16 acetylesterase was overproduced on lignocellulose. When the woody and nonwoody secretomes were compared, GH1 and GH3 β-glycosidases were more abundant on poplar and straw, respectively and, among less abundant proteins, VP2 was overproduced on straw, while VP3 was only found on poplar. The treated lignocellulosic substrates were analyzed by two-dimensional nuclear magnetic resonance (2D NMR), and a decrease of lignin relative to carbohydrate signals was observed, together with the disappearance of some minor lignin substructures, and an increase of sugar reducing ends.

**Conclusions:**

Oxidoreductases are strongly induced when *P. ostreatus* grows on woody and nonwoody lignocellulosic substrates. One laccase occupied the first position in both secretomes, and three more were overproduced together with one VP and one MnP, suggesting an important role in lignocellulose degradation. Preferential removal of lignin *vs* carbohydrates was shown by 2D NMR, in agreement with the above secretomic results.

**Electronic supplementary material:**

The online version of this article (doi:10.1186/s13068-016-0462-9) contains supplementary material, which is available to authorized users.

## Background

*Phanerochaete chrysosporium* (order Polyporales) has been the model lignin-degrading organism for more than two decades [1]. Due to the interest on lignin degradation/modification—as a key step for the industrial use of plant biomass for the production of cellulose, biofuels, and other chemicals [2]—this fungus was the first basidiomycete whose genome was sequenced [3]. *P. chrysosporium* belongs to the group of wood-rotting basidiomycetes known as white-rot fungi (due to the whitish color of decayed wood after a partial removal of lignin) [4]. More recently, the genomes of other Polyporales were sequenced, such as: (i) *Postia placenta* [5], as a model causing agent of the so-called brown-rot decay of wood (due to its brownish color after polysaccharide removal) [4]; and (ii) *Ceriporiopsis subvermispora* [6], a white-rot fungus of biotechnological interest due to its selective degradation of lignin [7]. With the availability of massive sequencing tools, many other Agaricomycotina genomes were sequenced up to a total of 126 available (on 31 September 2015) at the Mycocosm portal (http://www.genome.jgi.doe.gov/programs/fungi) of the DOE Joint Genome Institute (JGI) [8]. Using this genomic information, several recent studies have discussed the genes involved in lignocellulose decay in saprotrophic basidiomycetes often in combination with transcriptomic and secretomic analyses [9–15], although they were still largely based on Polyporales species.

*Pleurotus ostreatus* is the second edible mushroom worldwide, just after *Agaricus bisporus* [16] (two species of the order Agaricales). While Polyporales generally grow on woody substrates, *Pleurotus* and other members of the Agaricales naturally grow on wood, leaf litter, and/or other nonwoody lignocellulosic substrates (wood, sawdust, and wheat/rice straw being used for their commercial production). Moreover, some *Pleurotus* species are able to remove lignin selectively from nonwoody lignocellulosic materials [17], as reported for *C. subvermispora* growing on wood. These species have been investigated for the biological production of cellulose [18] and biofuels [19] from wheat straw, a largely available plant feedstock for lignocellulose biorefineries [20].

*Coprinopsis cinerea* [21] and *A. bisporus* [22, 23], two typical coprophilous and litter/humus decomposers, respectively, and *Laccaria bicolor* [24], a model mycorrhizogenous fungus, are three additional Agaricales whose genome sequences are available. However, these fungi, and some poor wood decayers recently sequenced [25], are of limited biotechnological interest since their lifestyles do not require a significant degradation/modification of lignin. In contrast, *Pleurotus* and other Agaricales species are efficient lignin degraders causing white-rot decay of lignocellulosic materials. Therefore, the genome of *P. ostreatus* was sequenced both as an important edible mushroom and as a new model white-rot fungus of the order Agaricales (after *P. chrysosporium* from Polyporales) being able to grow both on woody and nonwoody lignocellulosic materials. This ability is most probably due to the presence of a new arsenal of lignin-degrading enzymes, as shown after heterologously expressing and characterizing all the ligninolytic peroxidases from the three families mentioned below [26, 27] and two dye-decolorizing peroxidase (DyP) genes from its genome. From 2010, the annotated genome of *P. ostreatus* is available at JGI as two monokaryons, PC9 (http://www.genome.jgi.doe.gov/PleosPC9_1) and PC15 (http://www.genome.jgi.doe.gov/PleosPC15_2), obtained from a commercial dikaryon [28]. Monokaryotic PC9, showing the highest growth rate, was used in recent transcriptomic [29] and present secretomic studies.

Comparative genomics, ideally combined with biochemical studies, has provided important clues on lignocellulose decay by white-rot and brown-rot saprotrophic basidiomycetes, as well as on host interactions by mycorrhizogenous and pathogenic fungi. Two conclusions of these studies are: (i) the presence of genes of ligninolytic peroxidases—from the lignin peroxidase (LiP), manganese peroxidase (MnP), and/or versatile peroxidase (VP) families—in the genomes of all typical white-rot (i.e., ligninolytic) basidiomycetes and their absence from all the brown-rot (i.e., cellulolytic) fungal genomes, as well as from those of some poor wood rotters; and (ii) the widespread distribution of other genes contributing to the oxidative attack on lignocellulose, such as those of H_2_O_2_-generating oxidases and laccases, in the genomes of wood-rotting basidiomycetes [9, 10, 15, 25, 30]. However, transcriptomic and, especially, secretomic studies are expected to offer the final picture on the enzymatic mechanisms involved in the extracellular decay of lignin, and other lignocellulose constituents, by saprotrophic fungi. Such information has exponentially increased during the last years, as described in a recent review [31]. Interestingly, these studies have shown the variability of enzymes secreted by species with similar genomic contents when growing on the same plant substrates, evidencing the importance of secretomic analyses [32].

In the present study, the secretome of the model white-rot agaric *P. ostreatus* growing on woody (poplar wood) and nonwoody (wheat straw) lignocellulose was analyzed and compared with that from a glucose medium, with special emphasis on lignin-modifying enzymes (LMEs) and carbohydrate-active enzymes (CAZys). Secreted proteins were identified by nano-liquid chromatography coupled to tandem mass spectrometry (nLC-MS/MS) after trypsin hydrolysis, and their differential production discussed in the context of lignocellulose modification, which was analyzed using two-dimensional nuclear magnetic resonance (2D NMR) of the whole lignocellulosic samples at the gel state [33].

## Results

### Diversity of *P. ostreatus* proteins in the poplar, straw, and HAT secretomes

**Fig. 1:**
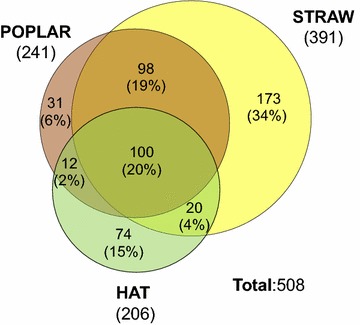
Venn diagram of total protein numbers in the *P. ostreatus* poplar, wheat straw, and HAT secretomes. See Fig. 3 for identification of the main (top-50) proteins in the poplar, straw, and HAT (glucose medium) secretomes (and Additional file 2 for the complete protein lists in each of the secretomes)

In order to understand the enzymatic mechanisms of lignin and plant polysaccharide degradation by *P. ostreatus*, the secretome of this white-rot (ligninolytic) fungus was analyzed by nLC-MS/MS of the total peptides from trypsin hydrolysis. With this purpose, the fungus (monokaryon PC9) was grown on a woody (poplar chips) and a nonwoody (wheat straw) lignocellulosic substrate (with distilled water as the only additive), and the diversity and relative abundance of the secreted proteins (after 21 days) compared with those found in a glucose medium (HAT). A total of 241, 391, and 206 extracellular proteins were identified in the poplar, wheat straw, and HAT fungal cultures, respectively, as summarized in Fig. 1, where the numbers of unique proteins (i.e., those only detected in one of the secretomes) are indicated together with those shared by the three secretomes or only by two of them (the complete lists of proteins in each of these cultures are included in Additional file 2: Tables S1, S2, S3, respectively).

The different proteins were functionally classified into the following seven types: (i) Oxidoreductases, including LMEs; (ii) CAZys; (iii) Esterases; (iv) Proteases; (v) Phosphatases; (vi) Other function proteins; and (vii) Unknown function proteins. The overall protein composition of the different cultures was similar, in terms of the types present, except for the minor phosphatase group that was absent from the HAT cultures. When the protein numbers in each of the above types were considered, without taking into account the abundance of each of them (see Additional file 1: Figure S1), the highest diversity of CAZy proteins (31 % of total proteins) was found in the HAT medium, while more oxidoreductases (21 %) were identified in the lignocellulose cultures. Moreover, 27 % of the wheat straw proteins showed “other” functions (compared with 14 % and 12 % in the HAT and poplar secretomes) and around 20 % of the proteins in each secretome had unknown functions.

### Abundance of the main protein types in the three *P. ostreatus* secretomes

**Fig. 2:**
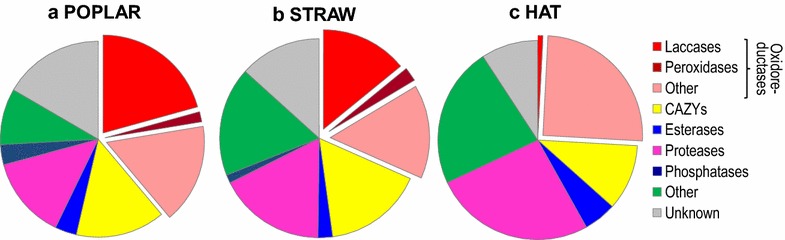
Relative abundance of the main protein types in the secretomes from three different media. **a** Poplar chips. **b** Wheat straw. **c** HAT medium. Additional information on the oxidoreductase presence is provided by showing the laccase, peroxidase, and other oxidoreductase abundances separately. The abundance of the different individual proteins was semiquantitatively estimated from their PSM number (see Additional file 2 for all PSM values)

Although *P. ostreatus* produced the same seven protein types in the three media and their diversity (in terms of protein numbers) only showed moderate changes as discussed above (Additional file 1: Figure S1), noteworthy differences were observed when a semi-quantitative analysis of the three secretomes was performed, based on the peptide-spectrum match (PSM) values of each of the proteins identified. As shown in Fig. 2, the relative abundance of oxidoreductases strongly increased from the HAT to the wheat straw and, especially, to the poplar cultures. This took place concomitantly with the marked decreases in the abundance of proteases and “other” proteins, and with a moderate increase of CAZys (the abundance of unknown proteins also increased). Interestingly, laccases are responsible for up to 21 and 14 % of total protein abundances in the poplar and straw cultures, respectively, but only 1 % in the HAT medium, supporting a role in lignocellulose degradation. The same tendency was observed for peroxidases, which were absent from HAT but present on both poplar and wheat straw, albeit with much lower abundances (2 % on both substrates) than laccases. In contrast, “other” oxidoreductases, including different oxidases, were significantly more abundant in the HAT (up to 25 % of total protein abundance) than in the poplar and straw cultures (15–16 %).

**Fig. 3:**
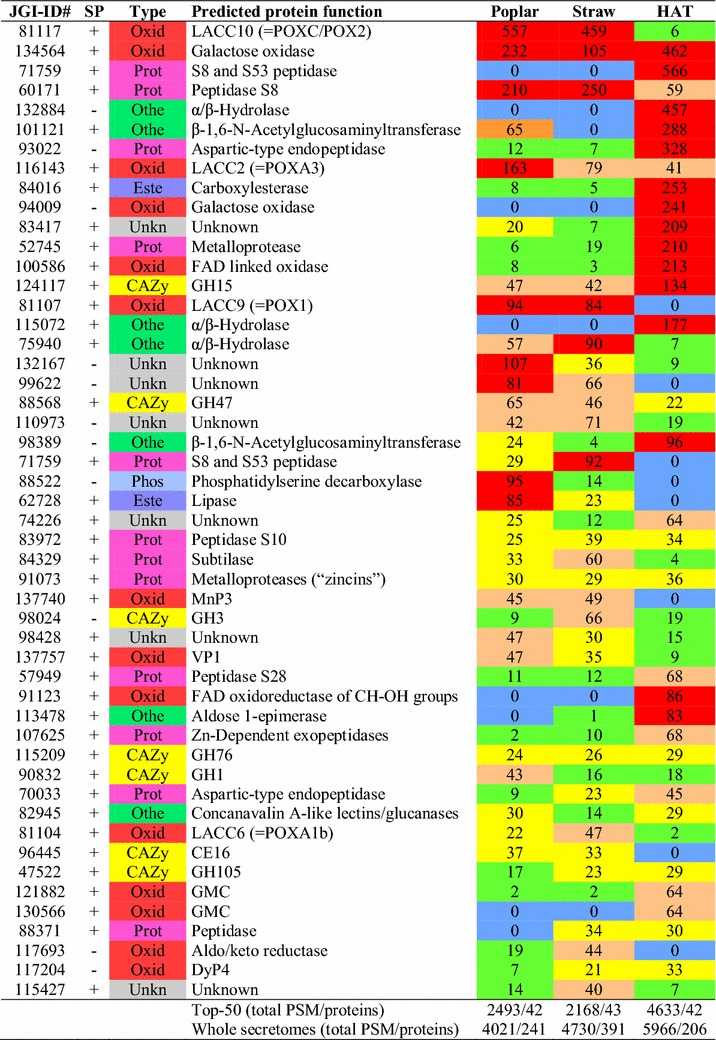
Fifty more abundant (top-50) proteins in the secretome of *P. ostreatus* growing on three different media. Semi-quantitative analysis based on PSM (peptide-spectrum match) values in the poplar, straw, and HAT (glucose medium) secretomes. The presence/absence of a predicted signal peptide (SP) is also indicated for the different proteins. The protein reference numbers, here and in the rest of the study and Additional file 2, correspond to the JGI Gene Catalog for *P. ostreatus* PC9. Abbreviations for protein types: *CAZy* carbohydrate-active proteins; *Este* esterases; *Othe* proteins with other functions; *Oxid* oxidoreductases; *Phos* phosphatases; *Prot* proteases; *Unkn* unknown function proteins

**Fig. 4:**
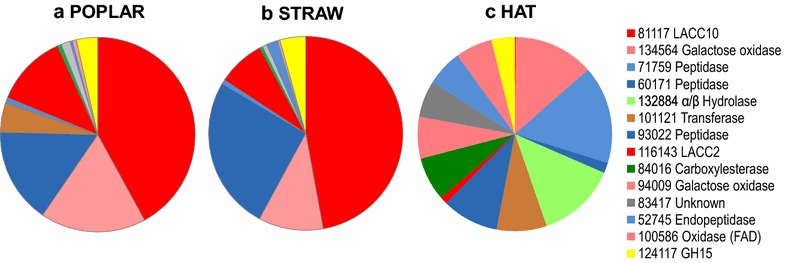
Relative abundance of the main (*14*) individual proteins in the secretomes from three different media. **a** Poplar chips. **b** Wheat straw. **c** HAT medium

The abundances of the 508 *P. ostreatus* individual proteins strongly varied (from 1 to 566 PSM values). Therefore, a more detailed analysis in the next sections focused on the 50 more abundant (top-50) proteins, whose references (JGI-ID #), type, predicted function, presence/absence of predicted signal peptide, and abundances (PSM values) are shown in Fig. 3. Although these 50 proteins are a small fraction of the total protein numbers, they represent 62, 46, and 78 % of the total protein abundances in the poplar, straw, and HAT secretomes, respectively. Interestingly, laccases and other LMEs, were among the main proteins in the lignocellulose secretomes. Although CAZys as a group were slightly more abundant in the lignocellulose cultures, the tendency is not general and some of them were more abundant in the HAT medium or did not show strong distribution differences. In the HAT culture, proteases, galactose oxidases, and α/β-hydrolase were among the most abundant proteins. Nonetheless, proteins with unknown function represented an important fraction of the top-50 proteins. The radical differences between the secretome from the HAT and lignocellulose cultures are illustrated in Fig. 4, where the relative abundances of the (14) main individual proteins discussed below are compared.

### *P. ostreatus* CAZys

CAZys are involved in the synthesis, metabolism, and transport of carbohydrates. Twenty-six percent of the 112 CAZy proteins identified were present in the three *P. ostreatus* secretomes, 16 % were shared by the poplar wood and wheat straw secretomes, and much lower numbers were shared by the poplar–HAT (4 %) and straw–HAT (7 %) secretomes (Additional file 1: Figure S2A). Finally, the HAT and wheat straw secretomes had higher number of unique CAZys (19–20 %) than the poplar secretome (only 8 %).

**Fig. 5:**
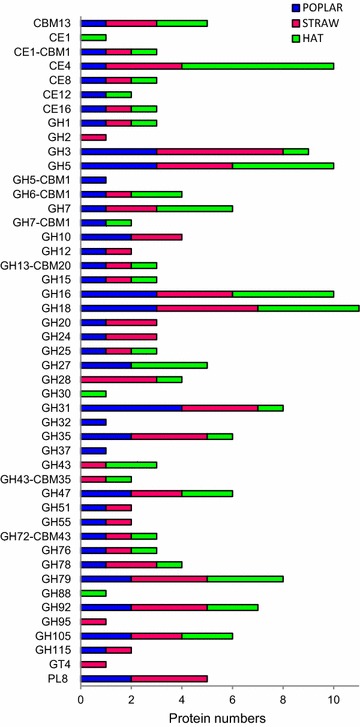
Diversity of CAZy proteins in the poplar, wheat straw, and HAT secretomes. The different families detected in each of the secretomes—including carbohydrate-binding modules (CBM), carbohydrate esterases (CE), glycoside hydrolases (GH), glycoside transferases (GT), and polysaccharide lyases (PL)—and number of proteins belonging to each family are indicated

When CAZy classification was considered, 38, 38, and 33 different families were identified in the secretome of *P. ostreatus* growing on poplar, wheat straw, and HAT, respectively, up to a total of 47 families (Fig. 5). Glycoside hydrolases (GHs) were the most widespread group of CAZys (with 26/27/21 families in the poplar/wheat–straw/HAT secretomes), followed by carbohydrate-binding modules (CBMs; with 7/6/7 families) and carbohydrate esterases (CEs; with 4/3/5 families). Finally, one polysaccharide lyase (PL) family was identified in the two lignocellulose cultures (not in HAT), and one glycosyltransferase (GT) family on wheat straw. Differences in the number of proteins in each CAZy family were observed with up to: (i) four GH5 and GH31 proteins in the poplar secretome; (ii) four GH18 and five GH3 proteins in the wheat straw secretome; and (iii) four GH5, GH7, GH16, and GH18, and six CE4 proteins in the HAT secretome. This can be related to duplication of GH7 (16 copies), GH16 (at least 14 copies), GH3 (11 copies), GH18 (at least 10 copies), CE4 (10 copies), and GH5 (eight copies) genes in the *P. ostreatus* genome [15, 25].

**Fig. 6:**
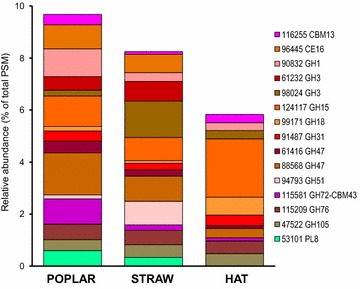
Relative abundance of main CAZy proteins in the secretomes from three different media. Distribution of the main glycoside hydrolases (GHs), glycosyltransferases (GTs), carbohydrate esterases (CEs), and cellulose-binding modules (CBM) in the poplar, wheat straw, and HAT secretomes (a total of 45 proteins)

The *P. ostreatus* secretomes not only differed in the CAZy diversity but also in their relative abundance as shown in Fig. 6 for the best represented proteins, most of them belonging to GH families (for all the CAZy proteins detected in the three secretomes, see Additional file 2). Among these proteins, we found two members of the GH3 family (JGI # 61232 and 98024), which exhibit β-glucosidase/β-xylosidase activities, and two members of the GH47 family (JGI-ID# 61416 and 88568), which exhibit α-mannosidase activity. GH3-98024 was the most abundant CAZy protein in wheat straw (1.4 % of total proteins) while it was less represented in the HAT and poplar cultures; and GH3-61232 showed a similar distribution. On the other side, GH47-88568 was the most abundant CAZy in poplar (1.6 % of total proteins) while it was less represented in the straw and HAT cultures, and a similar distribution was observed for GH47-61416. Moreover, the GH15 family, which includes glucoamylase activity, was represented by JGI-ID# 124117, the most abundant CAZy protein among the top-50 proteins (Fig.  3). In contrast with the other CAZys mentioned above, GH15-124117 showed the highest abundance in the HAT medium (2.2 % of total proteins). Proteins of families GH1 (including β-glycosidase activities), GH18 (including chitinase activity), GH31 (including α-glycosidase activities), GH51 (including endoglucanase/xylanase activities), GH76 (α-1,6-mannanase activity), and GH105 (unsaturated rhamnogalacturonyl/glucuronyl hydrolase activities) were also among the best represented members of the GH family.

Other CAZy groups also include well-represented proteins in the *P. ostreatus* secretomes, such as: (i–ii) families CE16 (showing acetylesterase activity; with JGI-ID# 96445) and PL8 (including hyaluronate lyase activity; with JGI-ID# 53101) only detected in the poplar and wheat straw cultures; and (iii) family CBM13 with JGI-ID# 116255 present in the three secretomes.

### *P. ostreatus* LMEs and peroxide-providing auxiliary oxidoreductases

The ligninolytic system of white-rot fungi includes extracellular laccases (phenol oxidases, POX), heme peroxidases, and oxidases generating hydrogen peroxide from the glucose/methanol/choline oxidase/dehydrogenase (GMC) and copper radical oxidase (CRO) superfamilies. However, only laccases and heme peroxidases of the LiP, MnP, and VP families are recognized as LMEs. The above oxidoreductases have been considered as auxiliary enzymes in CAZy families AA1 (laccases), AA2 (peroxidases), AA3 (GMC oxidases/dehydrogenases), and AA5 (CRO oxidases), respectively [34], but the term auxiliary enzymes is reserved here for the oxidases that provide the hydrogen peroxide required by ligninolytic peroxidases. Seventeen percent of the 103 oxidoreductase proteins identified were detected in the three *P. ostreatus* secretomes. Additionally, 27 % were shared by the poplar and wheat straw secretomes, and the HAT secretome only shared 2 % proteins with each of the lignocellulose secretomes (Additional file 1: Figure S2B). Finally, as in the case of CAZys, the wheat straw and HAT secretomes had higher numbers of unique oxidoreductases (32 and 14 %, respectively) than the poplar secretome (only 5 %).

**Fig. 7:**
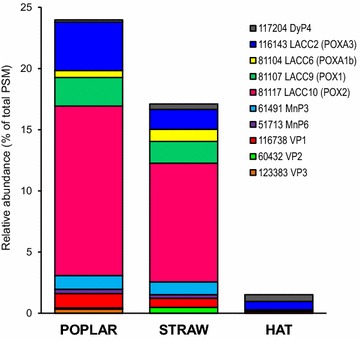
Relative abundance of main LMEs (peroxidases and laccases) in the secretomes from three different media. Distribution of the main peroxidases and laccases in the poplar, wheat straw, and HAT secretomes (a total of 11 proteins)

As shown in Fig. 7, LMEs were extremely abundant in the poplar (23 % of total protein abundance) and wheat straw (16 %) secretomes, while they were barely present in the HAT medium (only 1 %). Four laccase (LACC) proteins—LACC10 (JGI-ID# 81117), LACC2 (JGI-ID# 116143), LACC9 (JGI-ID# 81107), and LACC6 (JGI-ID# 81104)—were among the top-50 proteins (Fig. 3) with LACC10 occupying the first position due to its high abundance in the poplar/straw cultures (13.9/9.7 %). The poplar/wheat–straw abundances of LACC2 (4.0/1.6 %), LACC9 (2.3/1.8 %), and LACC6 (0.5/1.0 %) were also comparatively high. The four laccases showed similar abundances in the two lignocellulosic secretomes, with LACC9 being absent from the HAT cultures (Additional file 2).

Regarding class-II peroxidases, MnP3 (JGI-ID# 137740) and VP1 (JGI-ID# 137757) were the most abundant proteins, included in the top-50 (Fig. 3), although their relative abundances in the lignocellulose cultures (1.0–1.1 and 0.7–1.2 %, respectively) were largely below those of the four laccases. MnP6 (JGI-ID# 51713), VP2 (JGI-ID# 137766), and VP3 (JGI-ID# 123383) were also detected in the two lignocellulose cultures, with low abundances (0.3, 0.1–0.5, and 0–0.3 %, respectively). Only VP1 was identified in the HAT medium, although as a minor protein. DyPs contribute to degradation of lignin products, and *P. ostreatus* DyP4 (JGI-ID# 117204) was one of the few LME proteins in the HAT medium (0.6 % abundance), being also detected on poplar and wheat straw (Fig. 3).

Several oxidases would act synergistically with LME, providing the hydrogen peroxide required by peroxidases or reducing aromatic radicals formed by laccases. Among them, aryl-alcohol oxidase (AAO) was detected in the three secretomes (Additional file 2) but it was not among the top-50 proteins. Another two members of the GMC superfamily (JGI-ID# 121882 and 130566), and two related flavooxidases (JGI-ID# 100586 and 91123), were well represented in the HAT culture (1.1, 1.1, 3.6, and 1.4 % of all the proteins, respectively) (Fig. 3) but nearly absent from the lignocellulose cultures. In a similar way, two galactose oxidases (JGI-ID# 134564 and 94009) were among the main proteins in the HAT medium (7.7 and 4.0 %, respectively) being also present in the lignocellulose cultures (2.2–5.8 and 0 %, respectively).

### Other secreted proteins

Together with oxidoreductases and CAZys, proteases are another main type of proteins in the *P. ostreatus* secretomes, included among the top-50 (Fig. 3). Some of the main proteases showed a markedly differential distribution, with JGI-ID# 71759 being the most abundant protein in the HAT secretome (9.5 %) but completely absent from the lignocellulose cultures. The same was observed for a carboxylesterase (JGI-ID# 84016) with 32- and 50-fold lower relative abundance in the poplar and straw secretomes than in the HAT secretome, respectively. However, the opposite tendency was exhibited by three additional proteases (JGI-ID# 60171, 93022, and 52745) with much higher (4/4-, 27/47-, and 35/11-fold higher, respectively) abundances on poplar/wheat–straw than in the HAT medium.

Among those classified as “other”, three α,β-hydrolases were in the top-50 (Fig. 3), with two of them (JGI-ID# 132884 and 115072) being exclusive of the HAT secretome (132884 being the third more abundant protein in this culture), and the third one (JGI-ID# 75940) showing higher abundance in the lignocellulose cultures. Finally, differences were also observed in the main unknown proteins, with some of them being significantly more abundant in the lignocellulose cultures (such as JGI-ID# 132167 and 99622, with 2.7 % relative abundance) and others in the HAT medium (such as JGI-ID# 83417, attaining 3.5 %).

### Lignocellulose modification as shown by 2D NMR

**Fig. 8:**
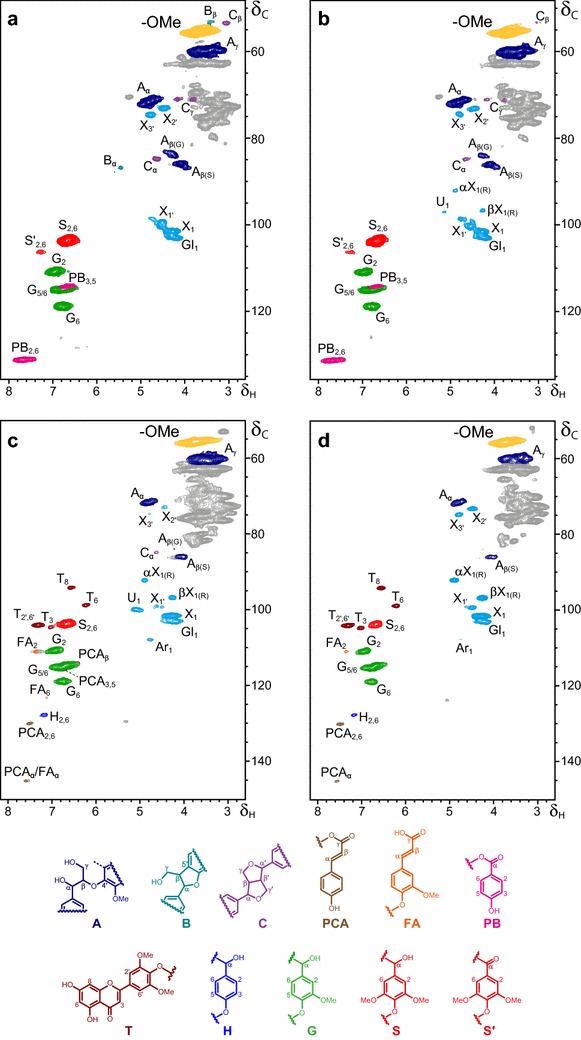
2D NMR of poplar wood (**a**, **b**) and wheat straw (**c**, **d**) treated with *P. ostreatus* (**b**, **d**) and controls (**a**, **c**). The formulae of the lignin and cinnamic acid structures, whose correlation signals are identified in the different spectra, are shown in the *bottom* of the figure: *A* β-O-4′ ether (*dark blue*); *B* phenylcoumaran (*turquoise*); *C* resinol (*purple*); *PCA* *p*-coumaric acid (*light brown*); *FA* ferulic acid (*orange*); *PB* *p*-hydroxybenzoate (*pink*); *T* tricin (*brown*); *H* *p*-hydroxyphenyl unit (*blue*); *G* guaiacyl unit (*green*); *S* syringyl unit (*red*); *S*′ α-oxidized syringyl unit (*red*). The prominent methoxyl signal (MeO, *yellow*) is also shown in the spectra, together with some selected carbohydrate correlation (C_1_–H_1_, C_2_–H_2_, and C_3_–H_3_) signals (*cyan*) corresponding to normal and acetylated xylan (X and X′, respectively), uronic acid (U), arabinan (Ar), and glucan units (Gl), α and β reducing ends in xylan (αX_1(R)_ and βX_1(R)_, respectively). *List of lignin (and related) signals* (δ_C_/δ_H_ ppm): 53.2/3.46, C_β_/H_β_ in phenylcoumarans (B_β_); 53.6/3.05, C_β_/H_β_ in resinols (C_β_); 55.5/3.66, C/H in methoxyls (−OMe); 71.1/4.16 and 3.77, C_γ_–H_γ_ in β–β′ resinols (C_γ_); 71.1–71.5/4.72–4.85, C_α_/H_α_ in β–O–4´ ethers (A_α_); 84.1/4.24, C_β_/H_β_ in β–O–4′ linked to a G unit (A_β(G)_); 84.9/4.59, C_α_/H_α_ in β–β′ resinols (C_α_); 86.0/4.08, C_β_/H_β_ in β–O–4′ ethers linked to a S unit (A_β(S)_); 86.7/5.41, C_α_/H_α_ in phenylcoumarans (B_α_); 94.2/6.56, C_8_/H_8_ in tricin (T_8_); 98.9/6.23, C_6_/H_6_ in tricin (T_6_); 103.8/6.68, C_2_/H_2_ and C_6_/H_6_ in syringyl units (S_2,6_); 104.1/7.30, C_2′,6′_/H_2′,6′_ in tricin (T_2′,6′_); 104.7/7.03, C_3_/H_3_ in tricin (T_3_); 106.2/7.29, C_2_/H_2_ and C_6_/H_6_ in α-oxidized syringyl units (S′_2,6_); 110.7/6.93, C_2_/H_2_ in guaiacyl units (G_2_); 111.0/7.28, C_2_/H_2_ in ferulic acid (FA_2_); 114.0/6.40, C_β_/H_β_ in *p*-coumaric acid (PCA_β_); 114.9/6.75, C_3,5_/H_3,5_ in *p*-hydroxybenzoic acid (PB_3,5_); 115.0/6.58–7.00, C_5/6_/H_5/6_ in guaiacyl units (G_5/6_); 118.9/6.75, C_6_/H_6_ in guaiacyl units (G_6_); 123.3/7.11, C_6_/H_6_ in ferulic acid (FA_6_); 127.8/7.18, C_2,6_/H_2,6_ in *p*-hydroxyphenyl units (H_2,6_); 130.1/7.50, C_2,6_/H_2,6_ in *p*-coumaric acid (PCA_2,6_); 131.2/7.65, C_2,6_/H_2,6_ in *p*-hydroxybenzoic acid (PB_2,6_); and 145.2/7.56, C_α_/H_α_ in *p*-coumaric acid and ferulic acid (PCA_α_ and FA_α_). Additional signals (in *cyan*) correspond to selected correlations of carbohydrate xylose (X), including α/β reducing ends (X_(R)_), acetylated xylose (X′), arabinose (Ar), glucose (Gl), and uronic acid (U) units (unassigned carbohydrate signals are in *gray*)

Structural analysis of the whole wood and straw samples, without the need for previous isolation of the lignin and polysaccharide fractions, was possible by swelling the milled material in deuterated dimethylsulfoxide (DMSO-*d*_*6*_) giving a gel-like material, which was directly analyzed under liquid 2D NMR conditions in heteronuclear single-quantum correlation (HSQC) experiments. The NMR spectra of the treated poplar wood and wheat straw are shown in Fig. 8b, d, while those of the corresponding uninoculated controls are shown in Fig. 8a, c. The formulae of the different structures identified are included in the bottom of Fig. 8.

The aromatic/unsaturated region of the spectra (δ_C_/δ_H_ 90–150/6–8 ppm) included the different correlations of the lignin: (i) *p*-hydroxyphenyl units (H, blue), only detected in the wheat straw; (ii) guaiacyl units (G, green); (iii) normal syringyl units (S, red); and (iv) Cα-oxidized syringyl units (S’, red), only detected in the poplar wood. Other aromatic/olefinic correlations corresponded to *p*-hydroxybenzoic acid (PB, magenta) in the poplar wood spectra, and tricin (T, brown), ferulic acid (FA, orange), and *p*-coumaric acid (PCA, light brown) in the wheat straw spectra. On the other hand, the aliphatic-oxygenated region of the spectra (δ_C_/δ_H_ 50–120/3–5 ppm) included the correlations of lignin side chains forming different substructures, such as: (i) β-O-4′ ethers (A dark blue); (ii) phenylcoumarans (B, turquoise); and (iii) resinols (C, purple). This region also includes the carbohydrate correlations of hemicellulose, since cellulose is silent under the present conditions. Among them, the anomeric carbon (C_1_) signals of normal and acetylated xylose (X and X′, respectively), arabinose (Ar), glucose (Gl), and uronic acid (U) units are indicated in cyan (including α- and β-xylose reducing ends), together with those of C_2_/C_3_-acetylated xylose units, while all the other carbohydrate correlations were not assigned on the spectra (gray). Finally, the prominent correlation of lignin methoxyls is also observed (OMe, yellow) in this region.

During the 21-day growth of *P. ostreatus* on poplar wood or wheat straw (resulting in 7–10 % weight loss with respect to the controls) some modification of the remaining lignocellulosic material was produced, as shown by 2D NMR of the treated samples and controls. The first observation from this comparison is the increased ratio between carbohydrate (estimated from the anomeric carbon signals) and lignin (estimated as the total H, G, S, and S’ signals) in poplar wood (from 1.2 to 1.6) and, especially, in wheat straw (from 3.0 to 4.9) revealing a preferential degradation of lignin. The differential decrease of lignin and carbohydrate signals agrees with the strongest decrease of (Klason) lignin in wheat straw (from 22 to 12 %, after deducting ashes). Monosaccharide analysis in the Klason hydrolyzates only revealed slight changes of the glucose/mannose/xylose/arabinose/galactose percentages in the treated wood (71.9/13.9/7.7/4.9/1.6) and straw (66.0/11.6/11.2/9.9/1.3) with respect to the corresponding controls (72.8/13.8/7.4/4.7/1.4 and 65.2/11.1/11.1/10.5/2.2, respectively). Simultaneously with the relative decrease of lignin, the amount of *p*-hydroxybenzoic acid per 100 lignin units increased in the treated poplar wood.

The modest delignification of poplar wood was accompanied by a low structural modification of the remaining lignin (and other wood components). In this way, the H:G:S:S′ ratio passed from 0:48:50:2 in the control to 0:46:54:1 in the treated wood, and the percentage of syringol and phenylcoumaran substructures per 100 lignin units passed from 6 to 4 % and from 2 to 0 %, respectively, while that of β-O-4′ ethers remains practically unchanged. However, the higher decrease of the lignin signals in wheat straw was accompanied by stronger modifications of: (i) the residual lignin H:G:S:S′ ratio, which passed from 3:57:40:0 in the control to 5:62:33:0 in the treated wheat straw; and (ii) the percentage of resinol substructures per 100 lignin units passed from 1.6 to 0 %, while that of β-O-4′ ethers was less significantly modified (no phenylcoumarans were found in wheat straw). Interestingly, the two *p*-hydroxycinnamic acids present in wheat straw were differentially degraded, and the ferulic content decreased (from 12 to 6 % of the lignin content) while the *p*-coumaric content increased (from 2 to 5 % of the lignin content). Finally, the flavonoid tricin seems to be specially recalcitrant since its content (referred to lignin) was twofold higher after the fungal treatment (passing from 8 to 22 %). Some changes in the xylan signals were also observed on both wheat straw and poplar wood, including the presence of stronger anomeric correlations (α/β X_1(R)_) corresponding to reducing ends.

## Discussion

### Secretomes of lignocellulose-decaying basidiomycetes

Studies on the proteome of wood-rotting basidiomycetes have increased during the last years in parallel with the increasing number of sequenced genomes, which enabled protein identification by tandem mass spectrometry using genomic databases (such as JGI Mycocosm). Since wood (and lignin polymer) decay is necessarily an extracellular process [35], secreted proteins have been generally analyzed in these studies.

*P. chrysosporium* was the first basidiomycete whose secretome was studied in carbon- and nitrogen-limited glucose media, and using/adding more complex carbon sources in/to liquid media, such as: (i) microcrystalline cellulose [36–38]; (ii) milled poplar wood [39, 40]; (iii) xylan/starch [41]; (iv) technical lignin [42]; and (v) other lignocellulosic substrates [43] (the two latter studies using quantitative proteomic techniques). The *P. chrysosporium* secretome was also analyzed during solid-state fermentation (SSF) of red oak wood [44, 45], black pine wood [46], and wheat straw [47].

Other white-rot fungi whose secretomes have been analyzed are: (i) *Pleurotus sapidus* growing in submerged and SSF lignocellulose cultures [48, 49]; (ii) *Phanerochaete carnosa* growing on microcrystalline cellulose in liquid medium, and on spruce chips under SSF conditions (compared with *P. chrysosporium*) [50]; (iii) *Ganoderma lucidum* during SSF of sugarcane bagasse [51]; (iv) *Trametes trogii* growing on poplar wood blocks [52]; (v) *Irpex lacteus* during wheat straw SSF [53] (compared *with P. chrysosporium* and *P. ostreatus*); (vi) *C. subvermispora* growing in liquid medium with microcrystalline cellulose and milled aspen (compared with *P. chrysosporium*) [6, 54]; (vii) *Phlebiopsis gigantea* growing on aspen and pine wood wafers [12]; and (viii) *Pycnoporus cinnabarinus* in (ligno)cellulose supplemented maltose liquid cultures, and as a SSF mixed secretome (from cultures on five different plant substrates) [13].

The secretomes of other lignocellulose-decaying basidiomycetes were also analyzed, including: (i) the brown-rotters *P. placenta* in liquid medium with cellulose or aspen/pine wood (compared with *P. chrysosporium*) [5, 39, 55] and colonizing poplar chips on malt agar [56], and *Serpula lacrymans* growing on pine wood [57]; and (ii) the coprophilous *C. cinerea* growing in glucose-peptone-yeast extract medium [58]. Finally, general comparisons of basidiomycete secretomes—including additional eleven white-rot and four brown-rot species and some poor wood decayers (such as *Schizophyllum commune*)—have been reported using milled-aspen liquid medium, with special emphasis on CAZy proteins [9, 11].

### Global analysis of the *P. ostreatus* secretome

A total of 508 different proteins were identified in the secretome of *P. ostreatus* growing with poplar wood, wheat straw, and glucose as carbon sources. This number is higher than reported in most of the studies cited above, which included up to 356 secreted proteins for *P. chrysosporium* and 413 proteins for *P. placenta* growing in glucose, aspen, and pine media [55], 168 proteins for a different (dikaryotic) *P. ostreatus* strain (during SSF of wheat straw) [53], and only 18 proteins in an early study of the *P. sapidus* secretome [48]. A higher number of basidiomycete-secreted proteins (near eight-hundred) were only reported in a recent secretomic study of *P. chrysosporium* growing on wood from three different poplar genotypes [40].

The secretome of *P. ostreatus* strongly varied, with only 20 % of the proteins being common to the three cultures analyzed. Interestingly, a similar additional percentage (19 %) was shared by the straw and poplar cultures, while the glucose culture only shared 2 and 4 % proteins with the poplar and wheat–straw cultures, respectively, revealing a strong and common effect of lignocellulose on the secreted proteins. More than half of the proteins were present only in one of the cultures, including 34 % on wheat straw, 15 % on glucose, and (only) 6 % on poplar wood. This reveals that colonization of wheat straw requires a high number of unique proteins in addition to those shared with the poplar culture (and the 20 % common to the three growth conditions). Moreover, strong differential production was observed for some of the shared proteins, as discussed below.

The above differences were analyzed considering the relative numbers and abundances of the main protein types. Most of them corresponded to the groups of oxidoreductases, CAZys (including plant cellulose/hemicellulose/pectin and fungal cell-wall degrading enzymes) or unknown function proteins, with the three types showing similar diversities (protein numbers) in the poplar and straw secretomes. However, in the HAT medium the diversity of CAZys was higher, although no (ligno)cellulosic substrate was present.

The differences are more remarkable when the abundances of each of the proteins were considered. In this way, it was shown that oxidoreductases (including LMEs), are by large the most abundant proteins in the two lignocellulose cultures, and less abundant in the glucose medium, where proteases and unknown proteins had larger, and CAZy slightly lower, abundances than in the lignocellulose cultures. Moreover, noteworthy differences in the different oxidoreductase “superfamilies” were observed, with peroxidases and especially multicopper oxidases (laccases) being abundant in the lignocellulose cultures but nearly absent from the glucose medium, where other oxidoreductases (including different oxidases) were more abundant. The unknown proteins, whose abundance was emphasized in early studies on wood-rotting fungal secretomes [38], still represent an important challenge in the present secretomic studies. Concerning glucose medium, the presence of soluble peptides (from yeast extract and casamino acids) is most probably related to the high protease levels observed. Moreover, the higher abundance of secreted proteins in the glucose cultures (~6000 total PSM, compared with 4000–5000 total PSM in the lignocellulose cultures) is most probably due to the shaken conditions used (compared with stationary lignocellulosic cultures) that promote protein secretion, as reported for example for extracellular chitinases [59].

### CAZy proteins in the *P. ostreatus* secretomes

Seven CAZy families (GH15, GH47, GH3, GH76, GH1, CE16, and GH105) were among the top-50 proteins in the *P. ostreatus* secretomes, but only one of them (CE16 acetylesterase) was clearly overproduced on lignocellulose (with respect to the glucose medium). When the woody and nonwoody lignocellulosic secretomes were compared, GH1 and GH3 (two β-glycosidases) were significantly more abundant in the poplar and wheat straw cultures, respectively. Among less abundant proteins, GH51 and GH35 were also more represented in wheat straw. Interestingly, the first CAZy in the top-50 proteins is glucoamylase GH15, being secreted in the three culture media. All the above CAZy families have been reported in the secretomes of other white-rot fungi growing under liquid and SSF conditions, several of them (e.g., GH3, GH5 or GH10) being overproduced in the presence of lignocellulosic substrates [12, 13, 38–40, 43, 50, 53].

Most families of “bulk carbohydrate” CAZys acting on polysaccharide backbones (such as GH5, GH6, GH7, GH10, and GH28) were found in the *P. ostreatus* secretomes, often represented by different proteins. However, the most abundant CAZys detected (such as GH1, GH3, and CE16) belong to the so-called “accessory” CAZy families, with GH51 endoglucanase/endoxylanase being the exception (1 % abundance in the wheat straw secretome). The above contrasts with other studies where endo-cellulases/xylanases (e.g., family GH10) are among the main proteins secreted by white-rot basidiomycetes (e.g., *P. chrysosporium*, *P. gigantea*, or *C. subvermispora*) when growing in wood-containing media [12, 40, 54]. The low levels of “cellulases” (from the GH5, GH6, and GH7 families) could be related to the selective degradation of lignin reported by some *Pleurotus* species [17]. On the other hand, no lytic polysaccharide monooxygenase (LPMO, former GH61 family) nor cellobiose dehydrogenase (CDH) proteins were detected in the secretome of *P. ostreatus* under the present growth conditions, although 18 LPMO and 1 CDH genes were annotated in the (PC9) genome. This contrast with the important role attributed to these enzymes, acting synergistically in cellulose degradation by some fungal species [60].

In addition to the above CAZys involved in degradation of plant polysaccharides, at least 28 CAZy proteins putatively contributing to the autolysis of fungal cell wall were identified. Seventy-five percent of them belong to families GH16 and GH18 (involved in β-glucan and chitin degradation, respectively) but members of the GH13, GH30 and GH72 families were also present. Chitinases (GH18) and β-glucanases (GH16) have been identified in the secretomes of other basidiomycetes [5, 11, 38, 43, 61]. Their diversity in the *P. ostreatus* secretome (GH18 was the CAZy family with the highest protein number) could be related to the need of recycling nutrients (by hyphal autolysis) in 3-week-old cultures. Hyphal lysis would be also related to the high levels of proteases involved in nitrogen recycling, as reported in the *P. chrysosporium* secretome [38].

### LMEs and other oxidoreductase proteins in the *P. ostreatus* secretomes

The *P. ostreatus* genome includes at least ten laccase genes [15, 25], together with seventeen peroxidase genes corresponding to one class-I peroxidase, nine class-II peroxidases, three heme-thiolate peroxidases (HTPs), and four DyPs [62]. After their heterologous expression, the class-II peroxidases have been identified as three VPs (being able to degrade model dimers and depolymerize lignin) and six MnPs (also showing Mn-independent activities) [27]. In parallel, two divergent DyP types have been identified with DyP4 being able to oxidize Mn^2+^ to Mn^3+^, as MnPs and VPs do [63]. At least four of the above ten laccases, the three VPs, two of the six MnPs, and DyP4 were secreted by *P. ostreatus* when growing on lignocellulosic substrates, as shown in the present secretomic study. The above results agree with a transcriptomic study of the same fungus [29], as well as with previous biochemical studies reporting enzymatic activities (without identifying the specific genes expressed) in *P. ostreatus* cultures grown on lignocellulosic substrates [64–68].

LACC10 is the main protein in the two lignocellulose secretomes, and LACC2, LACC9, and LACC6 are also among the top-50 proteins, together with VP1, MnP3, DyP4, and other seven oxidoreductases. These four laccases had been previously cloned, and reported as POX1 (LACC9), POX2 (LACC10), POXA1B (LACC6), and POXA3 (LACC2) [69–72]. However, only two peroxidases (VP2 and MnP3) of the five found in the secretome had been previously cloned as two MnPs [73, 74] with veratryl alcohol oxidation by VP2 being reported later [75], and the other enzymes were only known from the genome sequence [27]. Concerning the ligninolytic ability of the above enzymes, only VP has been reported to degrade (non-phenolic) lignin model dimers [27], but it has been also shown that laccases in the presence of redox mediators can perform similar reactions [76] and strongly degrade lignin in lignocellulosic materials [77, 78], in agreement with their high abundance in the lignocellulose cultures of *P. ostreatus*.

The above laccases, VP1 and MnP3 were significantly more abundant in the lignocellulose cultures, while oxidases of two different superfamilies—galactose oxidases from the CRO superfamily [79], and several members of the GMC superfamily [30]—were more abundant in the glucose culture. Among less represented proteins, VP2 was overproduced in wheat straw with respect to poplar wood, while VP3 was only found on poplar. It is worth mentioning that the poplar/wheat–straw abundance of LACC10 was one order of magnitude higher than that of the main CAZy protein in the lignocellulose cultures. Unexpectedly, AAO, the best known *Pleurotus* GMC [80], was a minor protein in the two lignocellulosic secretomes suggesting that other oxidases, such as galactose oxidase [81] occupying the second position among the top-50 proteins (just after LACC10), would contribute in H_2_O_2_ supply to the *P. ostreatus* peroxidases.

Laccases and MnPs have been reported in the secretomes of a series of wood-rotting basidiomycetes, such as *C. subvermispora*, *G. lucidum*, *I. lacteus*, *P. ostreatus*, *S. lacrymans*, and *T. trogii* [6, 51–53, 57]. Interestingly, in agreement with the present results, LACC10 has been reported as the main laccase isoenzyme induced by wheat straw extracts in *P. ostreatus* cultures [82, 83]. The model ligninolytic basidiomycete *P. chrysosporium* represents a remarkable exception to the above enzyme presence in secretomes, due to the absence of laccase genes in its genome [3]. Although some studies failed to detect ligninolytic peroxidases in (ligno)cellulose/lignin containing cultures of *P. chrysosporium* [42, 43], the presence of at least six different *P. chrysosporium* LiP and MnP isoenzymes has been reported in (carbon or nitrogen limited) glucose and (ligno)cellulose-containing media [38, 39], and the presence of LiP proteins was also claimed in SSF cultures [47]. Moreover, secretomic studies on the related *P. carnosa* suggested the presence of LiP and MnP in cellulose and wood-containing media [50], although conclusive evidence is still to be provided. Interestingly, one MnP has been reported as the main secreted protein in (3-day-old) wood cultures of *C. subvermispora* [54], a fungus with up to thirteen MnP genes [84]. In a similar way, VP proteins, which have been proposed to play in Agaricales (where no LiP genes have been still reported) the same role of LiP in Polyporales [27], had been detected in the secretomes of two *Pleurotus* species, together with several MnPs [48, 53], in agreement with the present results.

The most significant finding of the present secretomic study is the *P.ostreatus* overproduction of LMEs (including four laccases, one VP, and one MnP) when growing in lignocellulose-containing media. Although laccases and peroxidases have been reported in secretomic studies of several white-rot basidiomycetes, as discussed above, the overproduction levels were in most cases much more modest than those found here for *P. ostreatus*. One exception could be *P. cinnabarinus* that secretes one laccase (JGI-ID# 8672) as the main protein in some lignocellulose-based liquid and SSF cultures [13]. Another coincidence with the above *P. cinnabarinus* study is the detection of MnP proteins only in the lignocellulosic secretomes supporting their contribution to lignin decay, maybe through peroxidation reactions [85].

### Lignocellulose modification by secreted *P. ostreatus* enzymes

Interestingly, the above oxidoreductase overproduction could be correlated with the chemical modification of the lignocellulosic substrates shown by 2D NMR at the gel state, a new methodology helping lignocellulose pretreatment studies [33, 86]. These analyses revealed a preferential removal of lignin (estimated from its aromatic signals) with respect to polysaccharides (estimated from the anomeric carbon signals). This removal was accompanied by a decrease in the S/G ratio of the remaining lignin (especially in wheat straw) and by the complete disappearance of some minor substructures that accompany the main β-O-4′ ethers, such as phenylcoumarans in poplar and resinols in wheat straw (estimated by the specific aliphatic signals of their different side chains). The NMR results also show that: (i) syringyl units are easier to be degraded by the fungus, in agreement with their higher methoxylation degree (that lowers their redox potential); and (ii) resinols, and other minor lignin substructures disappeared during the fungal treatment. Lignin modification in the presence, or even in the absence, of added mediators has been reported for both VP [27, 87] and laccase [78, 88] using NMR and other techniques.

The HSQC spectra also showed four aromatic compounds that are naturally incorporated to the lignin polymer: (i–ii) tricin and ferulic acid forming ether linkages on wheat straw lignin (by radical condensation as normal monolignols do) [89, 90]; and (iii–iv) *p*-coumaric and *p*-hydroxybenzoic acids forming ester linkages on the Cγ-hydroxyl of wheat straw and poplar lignin units, respectively [91, 92]. The changes in the abundances of these compounds suggest that ferulic acid is preferentially removed by the overproduced *P. ostreatus* oxidoreductases with respect to lignin units (and carbohydrates), while *p*-coumaric acid, *p*-hydroxybenzoic acid, and tricin would be more recalcitrant toward the fungal attack. The differences in cinnamic acid removal may be related to the higher methoxylation degree of ferulic compared to *p*-coumaric acid enabling its oxidation by the secretome laccases (and improving peroxidase degradation) [93]. Finally, the appearance of sugar reducing ends in the spectrum of treated wood, and their increased intensities in the spectrum of treated wheat straw suggest a partial depolymerization of polysaccharides by CAZys.

## Conclusions

Although some differences were observed between the two substrates, the secretome of *P. ostreatus* growing both on poplar wood and wheat straw was characterized by a strong overproduction of LMEs with respect to glucose medium. These overproduced oxidoreductases included four laccases (LACC10 being the most abundant among the 434 different extracellular proteins identified in the lignocellulose cultures), one VP and one MnP. In contrast, CAZy proteins only showed slightly higher production in the lignocellulose cultures (with members of the GH15, GH47, GH3, GH76, GH1, CE16, and GH105 families among the top-50 proteins identified in the three secretomes). The above results agreed with the preferential removal of lignin from the two lignocellulosic substrates shown by the ratio between the lignin (aromatic) signals and the carbohydrate (anomeric) signals in the 2D NMR spectra of the whole treated materials at the gel state, which was accompanied by structural modification of the remaining lignin and carbohydrates.

## Methods

### *P. ostreatus* strain and genome

Monokaryotic *P. ostreatus* PC9 (CECT20311) was used in the present study. This strain was isolated (together with monokaryon PC15) from dikaryotic *P. ostreatus* N001 (CECT20600) [28]. Its genomic DNA sequence was obtained at JGI in a project coordinated by A.G. Pisabarro (Public University of Navarre, Spain). The resulting 35.6 Mbp assembly is predicted to include 12,206 genes (available for searching at http://www.genome.jgi.doe.gov/PleosPC9_1/PleosPC9_1.home.html).

### Comparative analysis of secretomes

Secretomic studies were performed on *P. ostreatus* cultures in glucose medium and on two different lignocellulosic substrates. Glucose cultures (triplicate) were grown in 1-L shaken (200 rpm) flasks with 200 mL (surface to volume ratio of 0.7 cm^−1^) of HAT medium [94] containing 10 g glucose, 0.2 g KH_2_PO_4_, 0.5 g MgSO_4_.7 H_2_O, 1 g casamino acids, 1 g yeast extract, 0.368 g ammonium tartrate, and 1 L of distilled water (sterilized at 120 °C for 20 min). Inocula consisted of 15 mL of homogenized actively growing mycelium from M7GY [82] liquid cultures (200 rpm). Lignocellulose cultures (triplicate) were grown on 10 g of chopped wheat (*Triticum aestivum*) straw or extractives-containing debarked poplar (*Populus alba*) small chips (particle size <4 mm in both cases) soaked with 70 mL of distilled water in 1-L flasks (surface to volume ratio of 1.9 cm^−1^) sterilized at 120 °C for 20 min, incubated under stationary conditions. Inocula consisted of 15 mL of homogenized mycelium from M7GY cultures. All the above cultures were maintained at 25 °C.

Cultures in the above media were grown for 21 days, triplicates were combined, filtered under vacuum, and the filtrates used for proteomic analyses, while the solid fraction (from the lignocellulose cultures) was used for the chemical analyses described in the next section. Total extracellular proteins in the filtrates were freeze-dried, resuspended in 10 mM tartrate (pH 5), impurities removed by a short polyacrylamide gel electrophoresis run, and stained by Colloidal Blue Kit (Invitrogen). The protein band was cut and destained using 50 mM ammonium bicarbonate in 50 % acetonitrile (ACN), reduced with 10 mM dithiothreitol for 30 min at 56 °C, alkylated with 55 mM iodoacetamide in the dark for 30 min at 24 °C, and digested with 12.5 ng·µL^−1^ trypsin in 50 mm ammonium bicarbonate, overnight at 30 °C. Peptides were extracted at 37 °C using 100 % ACN, and then 0.5 % trifluoroacetic acid, dried, cleaned using ZipTip with 0.6 μL C18 resin (Millipore), and reconstituted in 5 μL of 0.1 % formic acid in 2 % ACN.

Tryptic peptides were analyzed in an LTQ-Orbitrap Velos mass spectrometer (Thermo Scientific) coupled to a nanoEasy high-performance liquid chromatography equipment (Proxeon). Peptides were first trapped onto a C18-A1 ASY-Column 2 cm precolumn (Thermo Scientific), and then eluted onto a Biosphere C18 column (75 μm inner diameter, 15 cm long and 3 μm particle size) (NanoSeparations) using a 130 min gradient from 0–45 % buffer-B (buffer-A: 0.1 % formic acid in 2 % ACN; buffer-B: 0.1 % formic acid in pure ACN) at a flow rate of 250 nL.min^−1^. Mass spectra were acquired in the positive ion mode and data dependent manner selecting the 20 most intense ions for fragmentation using CID (collision induced dissociation). MS spectra (*m/z* 300–1600) were acquired in the Orbitrap with a target value of 1,000,000 at a resolution of 30,000 (at *m/z* 400) and MS2 spectra were acquired in the linear ion trap with a target value of 10,000 and normalized collision energy of 35 %. Precursor ion charge state screening and monoisotopic precursor selection were enabled. Singly charged ions and unassigned charge states were rejected. Dynamic exclusion was enabled with a repeat count of one and exclusion duration of 30 s.

Acquired spectra were searched against the *P. ostreatus* PC9 genomic database, downloaded from JGI (http://www.genome.jgi.doe.gov/PleosPC9_1/PleosPC9_1.download.html), using Sequest search engine through Proteome Discoverer (version 1.4). As for the search parameters, precursor and fragment mass tolerance were set to 10 ppm and 0.8 Da, respectively. Carbamidomethylation of cysteines was set as a fixed modification and oxidation of methionines was set as a dynamic modification. Two missed cleavages were allowed. Identified peptides were validated using Percolator algorithm with a *q*-value threshold of 0.01. The presence/absence of a signal peptide was predicted with SignalP 4.1 [95].

### NMR analyses of lignocellulose modification

The solid fraction from poplar wood and wheat straw treated with *P. ostreatus*, and from uninoculated controls kept (for 21 days) under the same conditions, were dried in an aeration oven at 65 °C until stable weight (to estimate weight losses during the treatments). The dried material was grounded in an IKA A10 cutting mill, and finely milled using a Fritsch Pulverisette six planetary mill at 400 rev·min^−1^ for 5 h (with 10 min breaks after every 10 min of milling) using a 500-mL agate jar and agate ball bearings (20 × 20 mm). Lignin content (as Klason lignin) was estimated as the residue after sulfuric acid hydrolysis of the samples according to Tappi test method T222 om-88 [96]. Neutral sugars in the same hydrolysates were analyzed by gas chromatography, after derivatization to their corresponding alditol acetates [97].

For NMR analysis, 100 mg of milled samples were swollen in DMSO-*d*_*6*_ and HSQC spectra were acquired at the gel state [33, 86, 98]. A Bruker AVANCE III 500 MHz spectrometer (Karlsruhe, Germany) fitted with a cryogenically cooled 5 mm TCI gradient probe with inverse geometry (proton coils closet to the sample) was used. The ^13^C-^1^H correlation experiment was an adiabatic HSQC experiment (using Bruker standard pulse sequence ′hsqcetgpsisp.2′; phase-sensitive gradient-edited-2D HSQC using adiabatic pulses for inversion and refocusing). Spectra were acquired from 10 to 0 ppm in F2 (^1^H) with 1000 data points for an acquisition time of 100 ms, an interscan delay (D1) of 1 s, 200 to 0 ppm in F1 (^13^C) with 256 increments (F1 acquisition time 8 ms) of 32 scans. The ^*1*^*J*_*CH*_ used was 145 Hz. Processing used typical matched Gaussian apodization in ^1^H and a squared cosine bell in ^13^C. The central DMSO peak was used as an internal reference (δ_C_/δ_H_ 39.5/2.49 ppm). The aromatic ^13^C-^1^H correlation signals of the different lignin units were used for estimation of composition in *p*-hydroxyphenyl (H), guaiacyl (G), syringyl (S) and C_α_-oxidized syringyl (S´) units, and the *p*-hydroxybenzoic acid (PB), *p*-coumaric acid (PCA), ferulic acid (FA), and tricin (T) contents were referred to total lignin (estimated as H + G+S + S´). The aliphatic ^13^C_α_-^1^H_α_ correlation signals of the β-O-4′ ether (A), phenylcoumaran (B), and resinol (C) side chains were used to estimate the relative abundances of the above substructures per aromatic unit. The intensity corrections introduced by the adiabatic pulse program permit us to refer the latter integrals to the previously obtained number of lignin units. Assignment of lignin (and hemicellulose) signals was based on previous wheat straw and hardwood NMR studies [89, 99–102].

## Additional files

10.1186/s13068-016-0462-9 Protein diversity and CAZy and oxidoreductase Venn diagrams in the three *P. ostreatus* secretomes analyzed.
10.1186/s13068-016-0462-9 Complete lists of proteins identified in the secretome of *P. ostreatus* growing on three different media.

## Abbreviations

AAO: aryl-alcohol oxidase
ACN: acetonitrile
CAZy: carbohydrate-active enzyme
CBM: carbohydrate-binding module
CDH: cellobiose dehydrogenase
CE: carbohydrate esterase
CRO: copper radical oxidases
DyP: dye-decolorizing peroxidase
GH: glycoside hydrolase
GMC: glucose/methanol/choline oxidases/dehydrogenases
HSQC: heteronuclear single-quantum correlation
LACC: laccase
LiP: lignin peroxidase
LME: lignin-modifying enzyme
LPMO: lytic polysaccharide monooxygenase
MnP: manganese peroxidase
nLC-MS/MS: nano-liquid chromatography-mass spectrometry/mass spectrometry
NMR: nuclear magnetic resonance
POX: phenol oxidase
PSM: peptide-spectrum match
VP: versatile peroxidase
