# Supplementary material for: A secretomic view of woody and nonwoody lignocellulose degradation by Pleurotus ostreatus
Source: Biotechnol Biofuels. 2016 Feb 29;9:49. doi: 10.1186/s13068-016-0462-9 (PMC4772462; doi:10.1186/s13068-016-0462-9)
Supplement: Supplementary file 1 — 10.1186/s13068-016-0462-9 Protein diversity and CAZy and oxidoreductase Venn diagrams in the three P. ostreatus secretomes analyzed. [file 13068_2016_462_MOESM1_ESM.pdf]

ADDITIONAL FILE 1: Supplemental figures

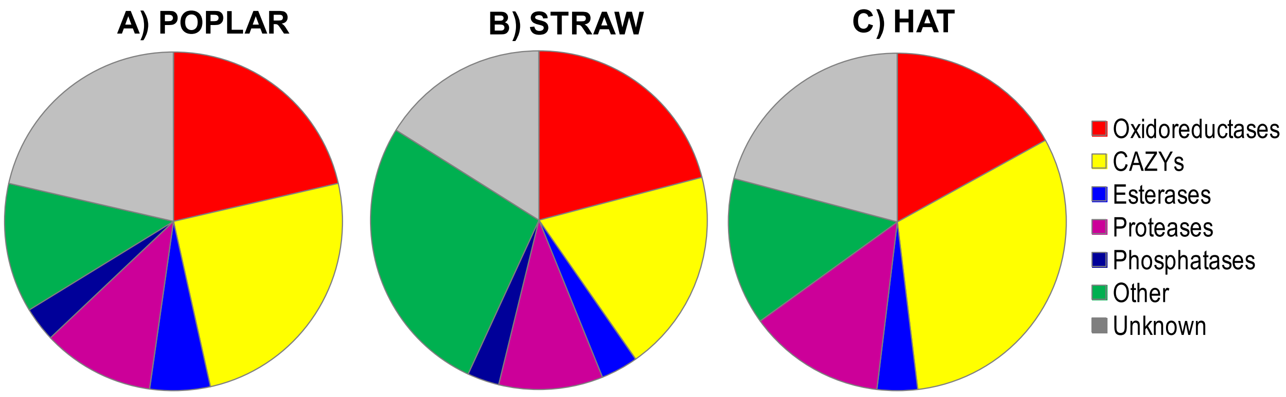

Figure S1. **Protein numbers (diversity) of the different functional types found in the secretomes from different media.** (**A**) Poplar chips. (**B**) Wheat straw. (**C**) HAT (glucose) medium (see Additional file 2: **Tables S1-S3** for the complete protein lists in each of the secretomes).

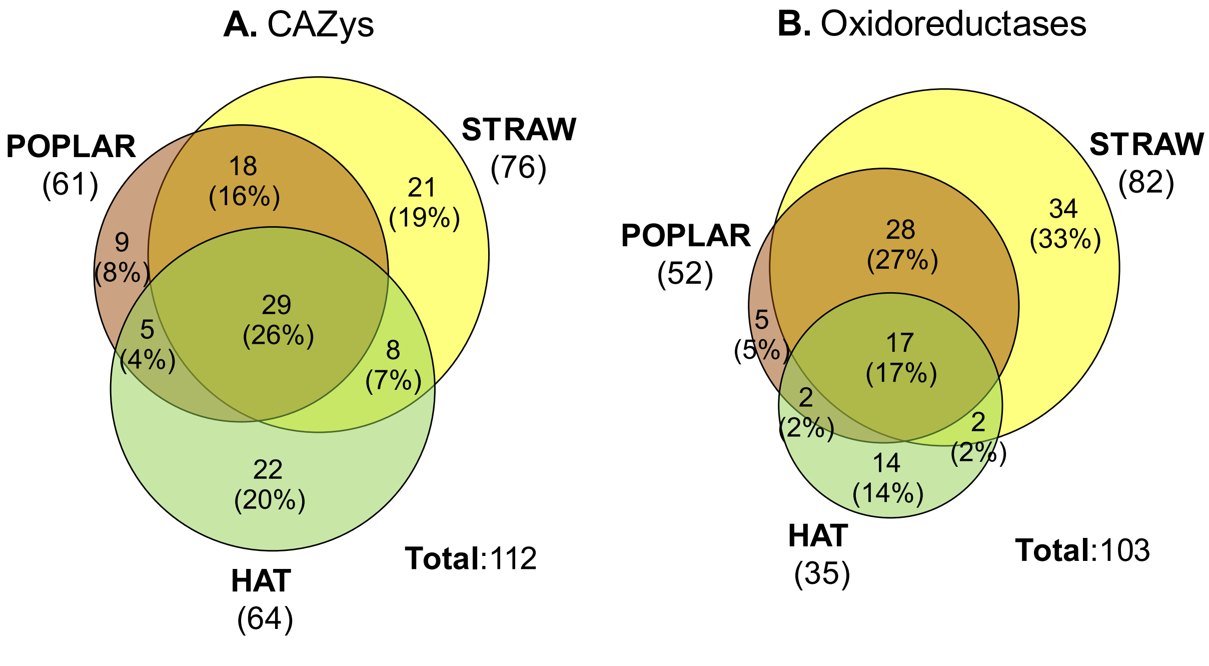

Figure S2. **Venn diagrams of CAZy (A) and oxidoreductase (B) numbers in secretomes.** The numbers of unique and shared proteins in the poplar wood, wheat straw and HAT secretomes are indicated (with percentages of total in parenthesis).
