# Supplementary material for: A secretomic view of woody and nonwoody lignocellulose degradation by Pleurotus ostreatus
Source: Biotechnol Biofuels. 2016 Feb 29;9:49. doi: 10.1186/s13068-016-0462-9 (PMC4772462; doi:10.1186/s13068-016-0462-9)
Supplement: Supplementary file 2 — 10.1186/s13068-016-0462-9 Complete lists of proteins identified in the secretome of P. ostreatus growing on three different media. [file 13068_2016_462_MOESM2_ESM.pdf]

ADDITIONAL FILE 2: Supplemental Tables S1-S3

Table S1. List of 241 proteins identified in the secretome of *P. ostreatus* growing on poplar wood[1]

JGI-ID#	Type[2]	Predicted protein function	PSM
81117	Oxid	LACC10	557
134564	Oxid	Galactose oxidase	232
60171	Prot	Peptidase S8	210
116143	Oxid	LACC2	160
132167	Unkn	Unknown	107
88522	Othe	Phosphatidylserine decarboxylase	95
81107	Oxid	LACC9	94
62728	Este	Lipase	85
99622	Unkn	Unknown	81
88568	CAZy	GH47	65
101121	Othe	β-1,6-N-Acetylglucosaminyltransferase	65
75940	Othe	α/β-Hydrolase	57
98428	Unkn	Unknown	47
124117	CAZy	GH15	47
137757	Oxid	VP1	47
137740	Oxid	MnP3	45
50262	Prot	Aspartyl protease	43
90832	CAZy	GH1	43
110973	Unkn	Unknown	42
62096	Othe	Amidohydrolase 2	39
115581	CAZy	GH72-CBM43	39
87572	Oxid	Cupredoxin	38
45030	Oxid	GMC	37
96445	CAZy	CE16	37
62166	Oxid	Galactose oxidase, central domain	36
84329	Prot	Subtilase	33
91073	Prot	Metalloproteases (“zincins”)	30
71759	Prot	S8 and S53 peptidase	29
117424	Unkn	Unknown	26
74226	Unkn	Unknown	25
83972	Prot	Peptidase S10	25
53101	CAZy	PL8	24
98389	Othe	β-1,6-N-Acetylglucosaminyltransferase	24
99623	Othe	Membrane attack complex	24
115209	CAZy	GH76	24
81104	Oxid	LACC6	22
82641	Prot	Serine-type peptidase	22
93955	Oxid	AAO	22
117427	Unkn	Unknown	22
61232	CAZy	GH3	21
82200	Unkn	Unknown	20
83417	Unkn	Unknown	20
117582	Oxid	Aldo/keto reductase	20

51352	Unkn	Unknown	19
72871	Unkn	Unknown	19
89221	Unkn	Unknown	19
117616	Oxid	GMC	19
117693	Oxid	Aldo/keto reductase	19
61416	CAZy	GH47	18
47522	CAZy	GH105	17
85683	CAZy	PL8	17
91487	CAZy	GH31	16
116255	CAZy	CBM13	16
116865	Unkn	Unknown	16
117123	Oxid	Flavodoxin/nitric oxide synthase	16
44086	CAZy	Barwin-like endoglucanases	15
55914	Othe	Amidase	15
82770	Othe	Phosphoglycerate mutase-like	15
115140	Prot	Aspartic-type endopeptidase	15
51713	Oxid	MnP6	14
115204	Oxid	Lipoxygenase	14
115427	Unkn	Unknown	14
123383	Oxid	VP3	14
59433	Oxid	GMC	13
62844	Prot	Peptidase S9	13
81199	Oxid	Glucose/ribitol dehydrogenase	13
85298	Prot	Cysteine-type endopeptidase	13
51118	Unkn	Unknown	12
89111	Prot	Peptidase S9	12
93022	Prot	Aspartic-type endopeptidase	12
45206	CAZy	GH6-CBM1	11
52629	Prot	Peptidase A1	11
56239	Este	Carboxylesterase	11
57949	Prot	Peptidase S28	11
96527	Prot	Serine carboxypeptidase	11
108781	Unkn	Unknown	11
114483	Othe	Fungal hydrophobin	11
114771	CAZy	GH7-CBM1	11
58999	Este	Survival protein SurE-like phosphatase/nucleotidase	10
62259	Unkn	Unknown	10
78300	Este	Ribonuclease T2	10
87860	Unkn	Unknown	10
61644	Othe	Lectin	9
69649	Oxid	GMC	9
70033	Prot	Aspartic-type endopeptidase	9
75056	Oxid	Thioredoxine	9
98024	CAZy	GH3	9
106062	Unkn	Unknown	9
109482	Othe	Thaumatin	9
114098	CAZy	GH55	9
114510	Oxid	GMC	9

115859	Unkn	Unknown	9
116309	Oxid	FAD binding oxidoreductase	9
130416	Unkn	Unknown	9
69654	Este	Endonuclease/exonuclease/phosphatase	8
84016	Este	Carboxylesterase	8
87982	Oxid	Glyceraldehyde-3-phosphate dehydrogenase	8
100586	Oxid	FAD linked oxidase	8
115319	Oxid	Flavodoxin/nitric oxide synthase	8
115623	CAZy	CE4	8
127085	Prot	Serine-type carboxypeptidase	8
47034	Prot	S8 and S53 peptidase	7
84903	Unkn	Unknown	7
84985	CAZy	GH92	7
88317	Prot	Serine-type carboxypeptidase	7
99171	CAZy	GH18	7
103254	Unkn	Unknown	7
109334	Othe	Transaldolase	7
114288	Este	Esterase/lipase	7
116582	Este	Survival protein SurE-like phosphatase/nucleotidase	7
117204	Oxid	DyP4	7
46733	Unkn	Unknown	6
52745	Prot	Metalloprotease	6
58838	Unkn	Unknown	6
67327	Unkn	Unknown	6
80285	Othe	Hydrophobic surface binding protein	6
94793	CAZy	GH51	6
100792	Unkn	Unknown	6
100990	Prot	Peptidase M	6
106719	Othe	Protein kinase	6
115424	Prot	PeptidaseM	6
116283	Unkn	Unknown	6
116829	Othe	Phosphoglucomutase	6
116926	CAZy	CE8	6
117865	Unkn	Unknown	6
50496	Este	Histidine acid phosphatase	5
65411	CAZy	GH130CBM20	5
69722	Unkn	Unknown	5
77872	Unkn	Unknown	5
82845	Este	Carboxylesterase	5
85083	Este	Metallophosphoesterase	5
86964	Oxid	FAD binding domain	5
87354	CAZy	GH35	5
89439	Prot	Peptidase	5
90219	CAZy	GH27	5
96680	CAZy	GH24	5
97269	Este	Carboxylesterase	5
110190	Este	Ribonuclease T2	5
115916	CAZy	GH5	5

116228	CAZy	GH5-CBM1	5
117154	Oxid	Coproporphyrinogen oxidase	5
117545	CAZy	GH25	5
127507	Oxid	GMC	5
55739	Othe	Oxalate decarboxylase	4
64440	Unkn	Unknown	4
71475	Este	Lipase	4
73763	Othe	Calmodulin	4
83320	CAZy	GH7	4
85079	CAZy	GH5	4
87509	CAZy	GH20	4
88922	Unkn	Unknown	4
91272	Unkn	Unknown	4
98296	Unkn	Unknown	4
114158	Unkn	Unknown	4
115088	Unkn	Unknown	4
115736	Unkn	Unknown	4
116181	CAZy	GH79	4
125205	Oxid	Methylmalonate-semialdehyde dehydrogenase (acylating)	4
126905	CAZy	GH78	4
133709	Unkn	Unknown	4
137766	Oxid	VP2	4
18313	CAZy	GH18	3
20207	Othe	α/β-Hydrolase	3
55443	Este	Esterase/lipase	3
59305	Othe	Guanyl-nucleotide exchange factor	3
61498	CAZy	GH16	3
70792	Unkn	Unknown	3
75413	Oxid	Aldo/keto reductase	3
82557	CAZy	GH31	3
82918	CAZy	GH31	3
84333	CAZy	GH92	3
86351	Oxid	Multicopper oxidase	3
87230	Este	Endonuclease/exonuclease/phosphatase	3
90132	Oxid	NAD(P)-binding	3
90895	Othe	Fumarylacetoacetate hydralase	3
91105	Unkn	Unknown	3
96691	CAZy	GH10	3
97290	Este	Lipase	3
101650	Unkn	Unknown	3
102212	CAZy	Barwin-like endoglucanases	3
107973	Oxid	FAD binding oxidoreductase	3
114053	CAZy	Barwin-like endoglucanases	3
114786	Othe	Methionine synthase II	3
116022	Othe	Enolase	3
116292	CAZy	GH79	3
116338	CAZy	GH37	3
117340	Oxid	Aldo/keto reductase	3

117351	CAZy	CE1-CBM1	3
121421	Este	Metallo dependent phosphatase	3
128131	CAZy	GH105	3
130214	CAZy	GH3	3
134433	CAZy	GH35	3
33068	Este	Endoribonuclease L-PSP	2
54694	Oxid	Dioxygenase	2
55745	Unkn	Unknown	2
57185	Este	Endonuclease/exonuclease/phosphatase	2
58910	Oxid	Aldo/keto reductase	2
62364	Unkn	Unknown	2
63765	CAZy	GH16	2
63775	Oxid	FAD binding catalytic	2
64335	Este	Lipase 3	2
69174	Unkn	Unknown	2
74063	Oxid	Uricase (urate oxidase)	2
75659	CAZy	GH5	2
77373	Oxid	Glyoxal oxidase	2
78619	CAZy	CE 12	2
82569	Unkn	Unknown	2
82943	Oxid	Amine oxidase	2
83261	Oxid	3-Isopropylmalate dehydrogenase	2
84216	Othe	Glyoxalase	2
84996	CAZy	GH12	2
85420	CAZy	GH18	2
88981	CAZy	GH31	2
91667	Oxid	Pyridine nucleotide-disulphide oxidoreductase	2
100288	Unkn	Unknown	2
107625	Prot	Zn-Dependent exopeptidases	2
112716	Este	Endoribonuclease L-PSP	2
114340	Othe	Chaperones HSP70/HSC70, HSP70 superfamily	2
115392	Unkn	Unknown	2
115613	Oxid	Superoxide dismutase (manganese)	2
115628	CAZy	CE4	2
115734	Unkn	Unknown	2
115754	Othe	Translation elongation factor EF-1 alpha/Tu	2
117082	Unkn	Unknown	2
117534	CAZy	GH27	2
117691	Unkn	Unknown	2
121882	Oxid	GMC	2
123302	Othe	α/β-Hydrolase	2
123331	Este	PL1	2
123396	Unkn	Unknown	2
125911	CAZy	GH10	2
126566	Este	Carotenoid ester lipase precursor	2
127463	CAZy	GH115	2
129600	Oxid	GMC	2
132186	CAZy	Barwin-like endoglucanases	2

62766	CAZy	GH32	1
67725	Prot	Metalloprotease (“zincins”)	1
70187	Prot	Zn-dependent exopeptidase	1
99730	Este	PLC-like phosphodiesterases	1
116971	Oxid	FAD binding domain	1
		Total	4021

[1]Semi-quantitative analysis based on PSM (peptide-spectrum match) values; [2]Protein types= CAZy, carbohydrate-active proteins; Este, esterases; Othe, proteins with other functions; Oxid, oxidoreductases; Phos, phosphatases; Prot, proteases; Unkn, unknown-function proteins

Table S2. List of 391 proteins identified in the secretome of *P. ostreatus* growing on wheat straw[1]

JGI-ID#	Type[2]	Predicted protein function	PSM
81117	Oxid	LACC10	459
60171	Prot	Peptidase S8	250
134564	Oxid	Galactose oxidase	105
71759	Prot	S8 and S53 peptidase	92
75940	Othe	α/β-Hydrolase	90
81107	Oxid	LACC9	84
116143	Oxid	LACC2	77
110973	Unkn	Unknown	71
98024	CAZy	GH3	66
99622	Unkn	Unknown	66
84329	Pep	Subtilase	60
137740	Oxid	MnP3	49
81104	Oxid	LACC6	47
88568	CAZy	GH47	46
117693	Oxid	Aldo/keto reductase	44
94793	CAZy	GH51	43
124117	CAZy	GH15	42
114483	Othe	Fungal hydrophobin	41
115427	Unkn	Unknown	40
83972	Prot	peptidase S10	39
115424	Prot	PeptidaseM	39
116865	Unkn	Unknown	38
114786	Othe	Methionine synthase II	37
61232	CAZy	GH3	36
132167	Unkn	Unknown	36
116022	Othe	Enolase	35
137757	Oxid	VP1	35
88371	Prot	Peptidase	34
96445	CAZy	CE16	33
98428	Unkn	Unknown	30
87982	Oxid	Glyceraldehyde-3-phosphate dehydrogenase	29
91073	Prot	Metalloproteases (“zincins”)	29
89439	Prot	Peptidase	28
117424	Unkn	Unknown	27
99623	Othe	Membrane attack complex component	26
106719	Othe	Protein kinase	26
115209	CAZy	GH76	26
69649	Oxid	GMC	25
75413	Oxid	Aldo/keto reductase	25
81199	Oxid	Glucose/ribitol dehydrogenase	25
47522	CAZy	GH105	23
62728	Este	Lipase	23
70033	Prot	Aspartic-type endopeptidase	23
137766	Oxid	VP2	23
45030	Oxid	GMC	22

109334	Othe	Transaldolase	22
117123	Oxid	Flavodoxin/nitric oxide synthase	22
134433	CAZy	GH35	22
117204	Oxid	DyP4	21
52745	Prot	Metalloprotease	19
93955	Oxid	AAO	19
115754	Othe	Translation elongation factor EF-1 alpha/Tu	19
117427	Unkn	Unknown	19
57580	Othe	Structural constituent of ribosome	18
82200	Unkn	Unknown	18
82641	Prot	Serine-type peptidase	18
87860	Unkn	Unknown	18
53101	CAZy	PL8	16
66181	Othe	Ubiquitin	16
82608	Othe	Amidase	16
86964	Oxid	FAD binding domain	16
90832	CAZy	GH1	16
117150	CAZy	S-adenosylhomocysteine hydrolase	16
127507	Oxid	GMC	16
74724	Othe	Structural constituent of ribosome	15
84985	CAZy	GH92	15
96680	CAZy	GH24	15
100288	Unkn	Unknown	15
115613	Oxid	Superoxide dismutase (manganese)	15
116375	Othe	Pyruvate decarboxylase	15
117582	Oxid	Aldo/keto reductase	15
126905	CAZy	GH78	15
50262	Prot	Aspartyl protease	14
51105	Prot	Subtilase	14
51713	Oxid	MnP6	14
55914	Othe	Amidase	14
57387	CAZy	β-N-Acetylhexosaminidase-like domain	14
82945	Othe	Concanavalin A-like lectins/glucanases	14
85083	Este	Metallophosphoesterase	14
87354	CAZy	GH35	14
88522	Othe	Phosphatidylserine decarboxylase	14
90132	Oxid	NAD(P)-binding	14
115916	CAZy	GH5	14
116829	Othe	Phosphoglucomutase	14
51118	Unkn	Unknown	13
56239	Este	Carboxylesterase	13
75730	Othe	PLP-dependent transferase	13
109482	Othe	Thaumatin	13
115319	Oxid	Flavodoxin/nitric oxide synthase	13
117616	Oxid	GMC	13
57949	Prot	Peptidase S28	12
70187	Prot	Zn-dependent exopeptidase	12
74226	Unkn	Unknown	12

91487	CAZy	GH31	12
101650	Unkn	Unknown	12
110190	Este	Ribonuclease T2	12
116926	CAZy	CE8	12
127085	Prot	Serine-type carboxypeptidase	12
47034	Prot	S8 and S53 peptidase	11
60199	Unkn	Unknown	11
61416	CAZy	GH47	11
63225	Oxid	NAD(P)-binding	11
75056	Oxid	Thioredoxine	11
84333	CAZy	GH92	11
84963	Othe	Structural constituent of ribosome	11
114340	Othe	Chaperones HSP70/HSC70, HSP70 superfamily	11
116481	Othe	Transaldolase	11
116582	Este	Survival protein SurE-like phosphatase/nucleotidase	11
117567	Oxid	Phosphogluconate dehydrogenase (decarboxylating)	11
130214	CAZy	GH3	11
51352	Unkn	Unknown	10
62844	Prot	Peptidase S9	10
69654	Este	Endonuclease/exonuclease/phosphatase	10
83261	Oxid	3-Isopropylmalate dehydrogenase	10
85063	Prot	Peptidase S28	10
85102	Oxid	Aldo/keto reductase	10
87509	CAZy	GH20	10
107625	Prot	Zn-Dependent exopeptidases	10
115073	CAZy	GH3	10
115581	CAZy	GH72-CBM43	10
117154	Oxid	Coproporphyrinogen oxidase	10
133203	Unkn	Unknown	10
55443	Este	Esterase/lipase	9
58710	CAZy	GH78	9
58838	Unkn	Unknown	9
70501	Oxid	Malate dehydrogenase	9
74063	Oxid	Uricase (urate oxidase)	9
84903	Unkn	Unknown	9
87572	Oxid	Cupredoxin	9
88317	Prot	Serine-type carboxypeptidase	9
89221	Unkn	Unknown	9
114250	Othe	Structural constituent of ribosome	9
114288	Este	Esterase/lipase	9
115934	Othe	Elongation factor 2	9
116181	CAZy	GH79	9
116206	Othe	Structural constituent of ribosome	9
116641	Oxid	Malate dehydrogenase	9
125205	Oxid	Methylmalonate-semialdehyde dehydrogenase (acylating)	9
58910	Oxid	Aldo/keto reductase	8
75359	Othe	Nucleoside-diphosphate kinase	8
78300	Este	Ribonuclease T2	8

98479	Othe	Structural constituent of ribosome	8
114214	Prot	Metallopeptidase	8
114369	CAZy	GT4	8
115821	Othe	Pyruvate kinase	8
117340	Oxid	Aldo/keto reductase	8
128131	CAZy	GH105	8
28894	CAZy	GH16	7
46733	Unkn	Unknown	7
52438	CAZy	GH20	7
52629	Prot	Peptidase A1	7
64171	Othe	Structural constituent of ribosome	7
73130	Othe	Translation initiation factor 3, subunit e (eIF-3e)	7
78036	Othe	Structural constituent of ribosome	7
83417	Unkn	Unknown	7
87230	Este	Endonuclease/exonuclease/phosphatase	7
93022	Prot	Aspartic-type endopeptidase	7
96186	Este	Inorganic pyrophosphatase	7
97269	Este	Carboxylesterase	7
100990	Prot	Peptidase M	7
114158	Unkn	Unknown	7
114566	Othe	Structural constituent of ribosome	7
115261	Othe	2-Methylcitrate dehydratase	7
116125	Trans	Phosphokerolase	7
116292	CAZy	GH79	7
116309	Oxid	FAD binding oxidoreductase acting on CH-OH groups	7
116545	CAZy	GH35	7
132795	Othe	Structural constituent of ribosome	7
44292	Unkn	Unknown	6
49436	Prot	Aspartic-type endopeptidase	6
49740	Othe	Structural constituent of ribosome	6
50496	Este	Histidine acid phosphatase	6
51750	Othe	Structural constituent of ribosome	6
54867	CAZy	GH430CBM35	6
62096	Othe	Amidohydrolase 2	6
69607	Othe	Structural constituent of ribosome	6
71475	Este	Lipase	6
71883	Othe	Rab GDI protein	6
82316	Othe	Chaperone	6
82770	Othe	Phosphoglycerate mutase-like	6
85079	CAZy	GH5	6
85685	CAZy	PL8	6
88827	Oxid	Catalase	6
89668	Othe	α/β-Hydrolase	6
89918	CAZy	Six-hairpin glycosidase-like	6
98296	Unkn	Unknown	6
99730	Este	PLC-like phosphodiesterases	6
103254	Unkn	Unknown	6
106062	Unkn	Unknown	6

114313	Prot	Dipeptidyl-peptidase III	6
114626	Oxid	Dehydrogenase (1-pyrroline-5-carboxylate)	6
115204	Oxid	Lipoxygenase	6
115859	Unkn	Unknown	6
117545	CAZy	GH25	6
117865	Unkn	Unknown	6
121422	Othe	Chaperonin Cpn60	6
123302	Othe	α/β-Hydrolase	6
130416	Unkn	Unknown	6
130544	CAZy	GH3	6
45547	CAZy	GH43	5
61644	Othe	Lectin	5
62166	Oxid	Galactose oxidase, central domain	5
64335	Este	Lipase 3	5
82943	Oxid	Amine oxidase	5
84016	Este	Carboxylesterase	5
85084	Este	Metallophosphoesterase	5
88761	Prot	Metalloendopeptidase	5
89478	CAZy	GH28	5
90953	CAZy	GH28	5
91272	Unkn	Unknown	5
93329	Unkn	Unknown	5
93757	Prot	Proteasome	5
96691	CAZy	GH10	5
99171	CAZy	GH18	5
104195	Othe	Structural constituent of ribosome	5
107973	Oxid	FAD binding oxidoreductase	5
113469	Oxid	Aldo/keto reductase	5
114605	Oxid	Amine oxidase	5
114996	Othe	Structural constituent of ribosome	5
115359	Othe	Phosphoenolpyruvate carboxykinase	5
115494	Prot	Subtilase	5
116166	Othe	ATP-citrate lyase	5
116255	CAZy	CBM13	5
117082	Unkn	Unknown	5
117351	CAZy	CE1-CBM1	5
117603	Oxid	Thioredoxin	5
117719	Othe	Glycine hydroxymethyltransferase	5
123396	Unkn	Unknown	5
47519	Unkn	Unknown	4
61877	CAZy	GH95	4
76499	Othe	Structural constituent of ribosome	4
77378	Oxid	NAD(P)-binding	4
80141	Oxid	Aldehyde dehydrogenase	4
80476	Othe	Peptidyl-prolyl cis-trans isomerase	4
82845	Este	Carboxylesterase	4
85683	CAZy	PL8	4
86091	Oxid	FAD binding catalytic	4

88922	Unkn	Unknown	4
90895	Othe	Fumarylacetoacetate hydralase	4
91667	Oxid	Pyridine nucleotide-disulphide oxidoreductase	4
95791	CAZy	GH5	4
98389	Othe	β-1,6-N-Acetylglucosaminyltransferase	4
104053	Othe	Structural constituent of ribosome	4
110670	Othe	Argininosuccinate synthase	4
111061	Oxid	NAD(P)-binding	4
114602	Prot	S8 and S53 peptidase	4
115756	Othe	Dienelactone hydrolase	4
116719	Othe	Structural constituent of ribosome	4
116886	Oxid	NAD(P)-binding	4
116971	Oxid	FAD binding domain	4
117335	Oxid	NAD(P)-binding	4
122614	Este	Carboxylesterase	4
122680	Othe	Structural constituent of ribosome	4
126430	Unkn	Unknown	4
133592	Othe	Hemopexin	4
133709	Unkn	Unknown	4
18313	CAZy	GH18	3
44086	CAZy	Barwin-like endoglucanases	3
44321	Unkn	Unknown	3
45197	Othe	Structural constituent of ribosome	3
48547	Unkn	Unknown	3
52165	Othe	Structural constituent of ribosome	3
54344	Othe	Structural constituent of ribosome	3
58999	Este	Survival protein SurE-like phosphatase/nucleotidase	3
59305	Othe	Guanyl-nucleotide exchange factor	3
62259	Unkn	Unknown	3
62364	Unkn	Unknown	3
63267	Othe	Mandelate racemase/muconate lactonizing enzyme	3
63765	CAZy	GH16	3
64533	Unkn	Unknown	3
70797	Othe	Structural constituent of ribosome	3
73723	Othe	Profilin/allergen	3
75589	Othe	F0F1-type ATP synthase	3
76283	Othe	Structural constituent of ribosome	3
82259	Othe	Structural constituent of ribosome	3
82569	Unkn	Unknown	3
84996	CAZy	GH12	3
85018	CAZy	GH28	3
85298	Prot	Peptidase	3
85420	CAZy	GH18	3
86351	Oxid	Multicopper oxidase	3
86370	CAZy	CE4	3
88121	Othe	Pyruvate carboxylase	3
88981	CAZy	GH31	3
90153	Oxid	NAD(P)-binding	3

100586	Oxid	FAD linked oxidase	3
101484	Oxid	Aldo/keto reductase	3
109912	Othe	WD40 repeat like	3
111867	Othe	Thiolase-like	3
112716	Este	Endoribonuclease L-PSP	3
114053	CAZy	Barwin-like endoglucanases	3
114149	Oxid	Lysine-ketoglutarate reductase/saccharopine dehydrogenase	3
114164	Othe	Transketolase	3
114415	Este	DNase I-like	3
114585	CAZy	GH92	3
114678	Unkn	Unknown	3
115140	Prot	Aspartic-type endopeptidase	3
116518	Prot	Endopeptidase	3
116749	Othe	Histone H4	3
116842	Othe	Structural constituent of ribosome	3
117301	Othe	β-1,6-N-Acetylglucosaminyltransferase	3
127463	CAZy	GH115	3
133387	Othe	Chaperonin clpA/B	3
134731	Oxid	FAD dependent oxidoreductase	3
22129	Unkn	Unknown	2
33068	Este	Endoribonuclease L-PSP	2
45206	CAZy	GH6-CBM1	2
48638	Othe	α/β-Hydrolase	2
48699	CAZy	GH79	2
49652	Othe	Kynurenine aminotransferase	2
49690	Othe	Purine phosphorylase, family 2	2
53822	Othe	DNA-binding HORMA	2
54160	Unkn	Unknown	2
54694	Oxid	Dioxygenase	2
55745	Unkn	Unknown	2
56050	Unkn	Unknown	2
59181	CAZy	GH18	2
59433	Oxid	GMC	2
62380	Oxid	FAD linked oxidase	2
65411	CAZy	GH130CBM20	2
65926	Othe	PLP-dependent transferases	2
68912	Oxid	Aldo/keto reductase	2
69174	Unkn	Unknown	2
70792	Unkn	Unknown	2
72169	Unkn	Unknown	2
72745	Othe	Aegerolysin	2
74322	Oxid	P450	2
74368	Unkn	Unknown	2
74745	CAZy	CBM13	2
75666	Oxid	Dehydrogenase	2
77045	Oxid	CCP	2
78083	Othe	Structural constituent of ribosome	2
78389	Othe	Structural constituent of ribosome	2

79147	Unkn	Unknown	2
81650	CAZy	GH10	2
82096	Oxid	Aldo/keto reductase	2
82362	Othe	26S Proteasome regulatory complex	2
82557	CAZy	GH31	2
82670	Prot	Peptidase M20	2
84785	Othe	Actin-related protein Arp2/3 complex, subunit ARPC4	2
85385	Othe	Nuclear pore complex, rNpl4 component (sc Npl4)	2
85526	CAZy	Cellobiohydrolase	2
85660	Othe	Structural constituent of ribosome	2
85832	Othe	Phosphoglycerate mutase-like	2
86008	Este	Lipase	2
87925	Oxid	GMC	2
88016	Othe	Translation initiation factor 3, subunit e (eIF-3e)	2
88069	Othe	Structural constituent of ribosome	2
88781	Prot	Peptidase	2
89635	Unkn	Unknown	2
89703	Oxid	FAD binding oxidoreductase	2
92630	Othe	α/β-Hydrolases	2
94291	CAZy	GH2	2
100231	CAZy	GH7	2
100542	Oxid	Alkyl hydroperoxide reductase	2
100792	Unkn	Unknown	2
105369	CAZy	GH24	2
107254	Unkn	Unknown	2
108781	Unkn	Unknown	2
109884	Othe	Structural constituent of ribosome	2
111904	Othe	Rho GDP-dissociation inhibitor	2
112873	Othe	Translation initiation factor 6	2
114098	CAZy	GH55	2
114108	Othe	Ribulose-phosphate binding barrel	2
114114	Othe	Small GTPase mediated signal transduction	2
114647	Othe	Chaperone (14-3-3 family)	2
114840	Prot	Proteasome	2
115029	Prot	Endopeptidase	2
115088	Unkn	Unknown	2
115315	Othe	Structural constituent of ribosome	2
115579	Unkn	Unknown	2
115623	CAZy	CE4	2
115628	CAZy	CE4	2
115734	Unkn	Unknown	2
115769	Othe	Structural constituent of ribosome	2
115805	Othe	Structural constituent of ribosome	2
116283	Unkn	Unknown	2
116474	Oxid	NAD(P)-binding	2
116962	Oxid	Aldo/keto reductase	2
117655	Othe	Structural constituent of ribosome	2
117691	Unkn	Unknown	2

120978	Oxid	Alcohol dehydrogenase	2
121759	Oxid	UDP-glucose/GDP-mannose dehydrogenase	2
121882	Oxid	GMC	2
122086	CAZy	GH7	2
126419	Oxid	Homogentisate 1,2-dioxygenase	2
130167	Oxid	Pyridine nucleotide-disulphide oxidoreductase	2
132590	Othe	Major facilitator superfamily	2
133172	Prot	Peptidase S33	2
134190	Oxid	NADH:flavin oxidoreductase	2
113478	Othe	Aldose 1-epimerase	1
		Total	4728

[1]Semi-quantitative analysis based on PSM (peptide-spectrum match) values; [2]Protein types= CAZy, carbohydrate-active proteins; Este, esterases; Othe, proteins with other functions; Oxid, oxidoreductases; Phos, phosphatase; Prot, proteases; Unkn, unknown-function proteins

Table S3. List of 206 proteins identified in the secretome of *P. ostreatus* growing on HAT (glucose) medium[1]

JGI-ID#	Type[2]	Predicted protein function	PSM
71759	Prot	S8 and S53 peptidase	566
134564	Oxid	Galactose oxidase	462
132884	Othe	α/β-Hydrolase	457
93022	Prot	Aspartic-type endopeptidase	328
101121	Othe	β-1,6-N-Acetylglucosaminyltransferase	288
84016	Este	Carboxylesterase	253
94009	Oxid	Galactose oxidase	241
100586	Oxid	FAD linked oxidase	213
52745	Prot	Metalloprotease	210
83417	Unkn	Unknown	209
115072	Othe	α/β-Hydrolase	177
124117	CAZy	GH15	134
98389	Othe	β-1,6-N-Acetylglucosaminyltransferase	96
91123	Oxid	FAD binding oxidoreductase acting on CH-OH groups	86
113478	Othe	Aldose 1-epimerase	83
57949	Prot	Peptidase S28	68
107625	Prot	Zn-Dependent exopeptidases	68
74226	Unkn	Unknown	64
121882	Oxid	GMC	64
130566	Oxid	GMC	64
60171	Prot	Peptidase S8	59
82943	Oxid	Amine oxidase	51
123302	Othe	α/β-Hydrolase	49
70033	Prot	Aspartic-type endopeptidase	45
99171	CAZy	GH18	41
116143	Oxid	LACC2	41
78300	Este	Ribonuclease T2	39
82653	Oxid	GMC	38
63775	Oxid	FAD binding catalytic	37
91073	Prot	Metalloproteases ("zincins")	36
83972	Prot	Peptidase S10	34
48638	Othe	α/β-Hydrolase	33
117204	Oxid	DyP4	33
88371	Prot	Peptidase	30
47522	CAZy	GH105	29
82945	Othe	Concanavalin A-like lectins/glucanases	29
115209	CAZy	GH76	29
91487	CAZy	GH31	24
51352	Unkn	Unknown	23
114605	Oxid	Amine oxidase	23
64738	Oxid	FAD binding oxidoreductase	22
88568	CAZy	GH47	22
84903	Unkn	Unknown	20
106719	Othe	Protein kinase	20
47622	Oxid	Amine oxidase	19

98024	CAZy	GH3	19
110973	Unkn	Unknown	19
116255	CAZy	CBM13	19
128249	Unkn	Unknown	19
62166	Oxid	Galactose oxidase, central domain	18
90832	CAZy	GH1	18
107973	Oxid	FAD binding oxidoreductase	18
56239	Este	Carboxylesterase	17
127085	Prot	Serine-type carboxypeptidase	16
98428	Unkn	Unknown	15
110190	Este	Ribonuclease T2	14
129600	Oxid	GMC	14
56494	Este	Carboxylesterase	13
87860	Unkn	Unknown	13
115859	Unkn	Unknown	13
122086	CAZy	GH7	13
44712	CAZy	GH88	12
88317	Prot	Serine-type carboxypeptidase	12
117824	Unkn	Unknown	12
65857	Oxid	GMC	11
85079	CAZy	GH5	11
114400	CAZy	GH5	11
115140	Prot	Aspartic-type endopeptidase	11
116181	CAZy	GH79	11
117545	CAZy	GH25	11
18313	CAZy	GH18	10
49304	Othe	Cyanovirin-N	10
89703	Oxid	FAD binding oxidoreductase	10
89918	CAZy	Six-hairpin glycosidase-like	10
94118	Othe	Cyanovirin-N	10
114288	Este	Esterase/lipase	10
117109	CAZy	GH5	10
117150	CAZy	S-adenosylhomocysteine hydrolase	10
76980	Unkn	Unknown	9
84985	CAZy	GH92	9
85083	Este	Metallophosphoesterase	9
87572	Oxid	Cupredoxin	9
99122	Unkn	Unknown	9
114602	Prot	S8 and S53 peptidase	9
116285	Unkn	Unknown	9
117351	CAZy	CE1-CBM1	9
132167	Unkn	Unknown	9
137757	Oxid	VP1	9
48699	CAZy	GH79	8
54867	CAZy	GH430CBM35	8
62844	Prot	Peptidase S9	8
82641	Prot	Serine-type peptidase	8
90315	Oxid	FAD binding oxidoreductase	8

102212	CAZy	Barwin-like endoglucanases	8
117340	Oxid	Aldo/keto reductase	8
123053	CAZy	CE4	8
70187	Prot	Zn-dependent exopeptidase	7
75940	Othe	α/β-Hydrolase	7
77872	Unkn	Unknown	7
87354	CAZy	GH35	7
89439	Prot	Peptidase	7
99623	Othe	Membrane attack complex component/perforin/complement C9	7
103254	Unkn	Unknown	7
115427	Unkn	Unknown	7
115581	CAZy	GH72-CBM43	7
115628	CAZy	CE4	7
116283	Unkn	Unknown	7
117229	Prot	Metallopeptidase (zinc binding)	7
45206	CAZy	GH6-CBM1	6
47034	Prot	S8 and S53 peptidase	6
51118	Unkn	Unknown	6
51203	CAZy	CBM13	6
58710	CAZy	GH78	6
61416	CAZy	GH47	6
71475	Este	Lipase	6
81117	Oxid	LACC10	6
84333	CAZy	GH92	6
85084	Este	Metallophosphoesterase	6
87575	Prot	S8 and S53 peptidase	6
105520	Othe	β-Lactamase	6
116309	Oxid	FAD binding oxidoreductase acting on CH-OH groups	6
128131	CAZy	GH105	6
63218	Oxid	NADH Dehydrogenase	5
69649	Oxid	GMC	5
74704	Unkn	Unknown	5
78619	CAZy	CE 12	5
85343	Unkn	Unknown	5
88908	Oxid	GMC	5
90219	CAZy	GH27	5
117851	Unkn	Unknown	5
132563	CAZy	GH16	**5**
133709	Unkn	Unknown	5
54071	CAZy	Barwin-related endoglucanase	4
60707	CAZy	GH18	4
82770	Othe	Phosphoglycerate mutase-like	4
82810	CAZy	CE16	4
83849	CAZy	GH7	4
84329	Pep	Subtilase	4
88786	CAZy	GH30	4
117056	Unkn	Unknown	4
117301	Othe	β-1,6-N-Acetylglucosaminyltransferase	4

117516	Unkn	Unknown	4
44321	Unkn	Unknown	3
53421	Unkn	Unknown	3
58838	Unkn	Unknown	3
65411	CAZy	GH130CBM20	3
67327	Unkn	Unknown	3
68673	Oxid	Aldo/keto reductase	3
69174	Unkn	Unknown	3
70356	CAZy	CE4	3
74732	Unkn	Unknown	3
76006	Othe	Yeast cell wall synthesis KRE9KNH1	3
82608	Othe	Amidase	3
84350	Oxid	Glyoxal oxidase	3
85298	Prot	Cysteine-type endopeptidase	3
86577	Unkn	Unknown	3
88922	Unkn	Unknown	3
89111	Prot	Peptidase S9	3
92243	Othe	GrpE nucleotide exchange factor	3
93955	Oxid	AAO	3
96527	Prot	Serine carboxypeptidase	3
109117	Othe	Ubiquitin and ubiquitin-like proteins	3
112290	CAZy	CE1	3
112745	CAZy	CE4	3
114053	CAZy	Barwin-like endoglucanases	3
114158	Unkn	Unknown	3
114510	Oxid	GMC	3
115916	CAZy	GH5	3
116926	CAZy	CE8	3
117534	CAZy	GH27	3
117616	Oxid	GMC	3
132288	CAZy	Barwin-related endoglucanase	3
20650	Unkn	Unknown	2
28894	CAZy	GH16	2
43698	CAZy	GH6-CBM1	2
46813	Othe	Thaumatin, pathogenesis-related	2
54605	Este	Ribonuclease T 2	2
61868	CAZy	GH27	2
62096	Othe	Amidohydrolase 2	2
62259	Unkn	Unknown	2
64533	Unkn	Unknown	2
80522	Este	Lipase 3	2
81104	Oxid	LACC6	2
82200	Unkn	Unknown	2
85018	CAZy	GH28	2
85420	CAZy	GH18	2
87754	CAZy	GH16	2
89571	Oxid	FAD linked oxidase,	2
91578	CAZy	GH43	2

97623	CAZy	GH43	2
108781	Unkn	Unknown	2
110578	Unkn	Unknown	2
114415	Este	DNase I-like	2
114637	Prot	Serine-type peptidase	2
114771	CAZy	GH7-CBM1	2
115573	Othe	Transcription initiation factor TFIID	2
115623	CAZy	CE4	2
116292	CAZy	GH79	2
116829	Othe	Phosphoglucomutase	2
116877	Unkn	Unknown	2
117023	Othe	Isomerase YbhE	2
117142	Unkn	Unknown	2
117317	Unkn	Unknown	2
125794	CAZy	CE4	2
129907	Prot	Caspase	2
		Total	5966

[1]Semi-quantitative analysis based on PSM (peptide-spectrum match) values; [2]Protein types= CAZy, carbohydrate-active proteins; Este, esterases; Othe, proteins with other functions; Oxid, oxidoreductases; Prot, proteases; Unkn, unknown-function proteins
